# Relationship between socioeconomic status and hypertension incidence among adults in southwest China: a population-based cohort study

**DOI:** 10.1186/s12889-024-18686-5

**Published:** 2024-05-02

**Authors:** Tao Luo, Shenrong Lin, Wenying Zhang, Xuejiao Li, Yiying Wang, Jie Zhou, Tao Liu, Guofeng Wu

**Affiliations:** 1 Department of Emergency, The Affiliated Hospital of Guizhou Medical University, Guizhou Medical University, Guiyang, 550004 China; 2 School of Basic Medical Sciences, Guizhou Medical University, Guiyang, 550025 China; 3 Medical College, Guizhou University, Guiyang, 550025 China; 4 Clinical College of Guizhou Medical University, Guiyang, 550004 China; 5 School of Public Health, the key Laboratory of Environmental Pollution Monitoring and Disease Control, Ministry of Education, Guizhou Medical University, Guiyang, 550025 China; 6 Guizhou Province Centre for Disease Control and Prevention, 101 Bageyan Road, Yunyan District, Guiyang City, Guizhou Province China

**Keywords:** Socioeconomic status, Hypertension, Cohort study, Hypertension prevention, Southwest China

## Abstract

**Purpose:**

To investigate the correlation between socioeconomic status (SES) and the incidence of hypertension among adults aged 18 or above in southwest China.

**Methods:**

A multistage proportional stratified cluster sampling method was employed to recruited 9280 adult residents from 12 counties in southwest China, with all participants in the cohort tracked from 2016 to 2020. The questionnaire survey gathered information on demographics, lifestyle habits, and household income. The physical exam recorded height, weight, and blood pressure. Biochemical tests measured cholesterol levels. The chi-square test was employed to assess the statistical differences among categorical variables, while the Cox proportional hazards regression model was applied to evaluate the association between socioeconomic status (SES) and the incidence of hypertension.

**Results:**

The finally effective sample size for the cohort study was 3546 participants, after excluding 5734 people who met the exclusion criteria. Adults in the highest household income group had a significantly lower risk of hypertension compared to those in the lowest income group (HR = 0.636, 95% CI: 0.478–0.845). Besides, when compared to individuals in the illiterate population, the risk of hypertension among adults with elementary school, junior high school, senior high school and associate degree educational level decreased respectively by 34.4% (HR = 0.656, 95%CI: 0.533–0.807), 44.9% (HR = 0.551, 95%CI: 0.436–0.697), 44.9% (HR = 0.551, 95%CI: 0.405–0.750), 46.1% (HR = 0.539, 95%CI: 0. 340–0.854). After conducting a thorough analysis of socioeconomic status, compared with individuals with a score of 6 or less, the risk of hypertension in participants with scores of 8, 10, 11, 12, and greater than 12 decreased respectively by 23.9% (HR = 0.761, 95%CI: 0.598–0.969), 29.7% (HR = 0.703, 95%CI: 0.538–0.919), 34.0% (HR = 0.660, 95%CI: 0.492–0.885), 34.3% (HR = 0.657, 95%CI: 0.447–0.967), 43.9% (HR = 0.561, 95%CI: 0.409–0.769).

**Conclusion:**

The findings indicate a negative correlation between socioeconomic status and hypertension incidence among adults in southwest China, suggesting that individuals with higher socioeconomic status are less likely to develop hypertension.

**Supplementary Information:**

The online version contains supplementary material available at 10.1186/s12889-024-18686-5.

## Introduction

Hypertension has emerged as a significant public health concern on a global scale, with an estimated1.39 billion individuals affected and over 10.8 million deaths attributed to the condition annually [1, 2]. In China alone, there are more than 245 million adults diagnosed with hypertension, resulting in medical expenditures exceeding 40 billion yuan per year [3, 4]. Prior research has indicated that in 2017, the nationwide death toll attributed to hypertension reached 2.54 million, with years of life lost (YLL) due to premature mortality from hypertension at 3154.3 per 100,000 individuals, and years lost due to disability (YLD) at 428.0 per 100,000 individuals [5–7].

The biopsychosocial (BPS) model [8] has highlighted that the development of hypertension is solely determined by biological factors such as obesity [9, 10] and blood lipid levels [11], but can also be influenced by behavioral patterns, lifestyle choices [12–14], and external environmental factors including economic status, level of education, professional and other factors [7, 15]. There is a growing awareness among individuals that socioeconomic status (SES) plays a substantial role in the occurrence of hypertension, particularly in light of the rapid evolution of social economies. SES, which encompasses factors such as household income, educational attainment, and occupation status, serves as a measure of an individual's professional background and economic and social standing in relative to others [16, 17]. Interestingly, our research has revealed a paradoxical relationship between SES and hypertension prevalence in developed nations, where SES is inversely associated with the incidence of hypertension, while contrasting findings are observed in developing countries.

Interestingly, we found a paradoxical phenomenon that SES is inversely associated with the incidence of hypertension in mostly developed countries [18, 19], while contrasting findings are observed in developing nations. Addo et al. [20] conducted a study which revealed a positive correlation between socioeconomic status (SES) and the prevalence of hypertension in Ghana, while Fateh et al. [21] found that individuals with low SES had a higher prevalence of hypertension in Iran. Moreover, cross-sectional data from Trinidad and Tobago [22] indicated a lack of significant relationship between SES and hypertension. These findings suggested that the association between SES and the prevalence of hypertension varies in different countries. Therefore, it holds significant scientific value to investigate the correlation between SES and hypertension within the Chinese population, offering theoretical insights for the prevention and management of hypertension from the perspective of SES.

## Materials and methods

### Ethics statement

The study was approved by the Research Ethics Committee of Guizhou Provincial Center for Disease Control and Prevention (ethical approval number: S2017-02), and conducted in accordance with the Declaration of Helsinki. All participants signed an informed consent for participating in the study.

### Study population

This study adopted a multistage proportional stratified cluster sampling method. A total of 9280 adult people from 12 counties in the southwest China were recruited at baseline 2010 to 2012. Participants were required to meet the following inclusion criteria: (1) age 18 years or above; (2) living in the study regions for more than six months; (3) completing a questionnaire and taking blood samples.

**Fig. 1:**
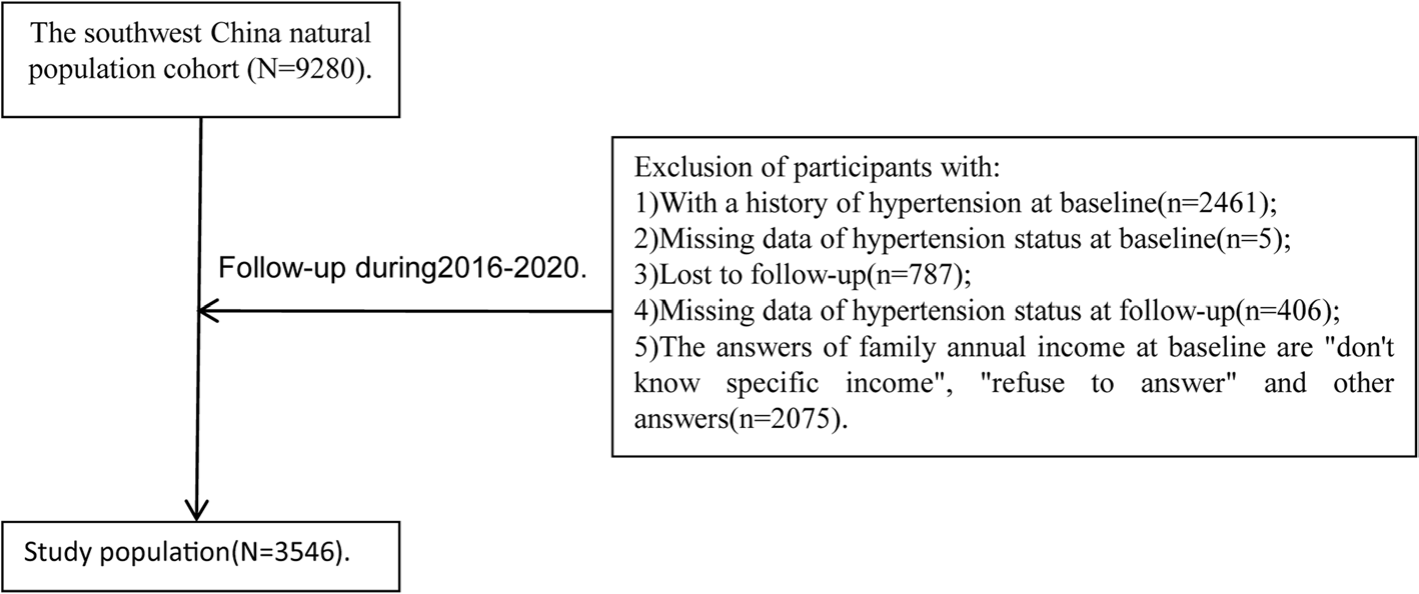
The flowchart of research object screening

The entire cohort was followed up from 2016 to 2020 We excluded 5734 participants who met the following exclusion criteria: (1) with a history of hypertension at baseline; (2) missing the data of hypertension status at baseline; (3) lost to follow-up; (4) missing the data of hypertension status at follow-up; (5) the answer of family annual income at baseline are “don’t know specific income”, “refuse to answer” and other answer. Consequently, a total of 3546 participants were eligible for the analysis. The flowchart of research object screening was shown in Fig. 1.

### Physical examination

Participants’ height, weight and blood pressure were measured, with body mass index (BMI) calculated by dividing body weight in kilograms (kg) by the square of height in meters (m^2^), based on the Criteria of the Ministry of Health, China [23, 24]. Participants were categorized into underweight (BMI < 18.5 kg/m^2^), normal weight (18.5 kg/m^2^ ≤ BMI < 24.0 kg/ m^2^), overweight (24.0 kg/m^2^ ≤ BMI < 28.0 kg/m^2^) and obesity (24.0 kg/m^2^ ≤ BMI < 28.0 kg/m^2^) based on their BMI. Blood pressure was measured following a 30-min rest period, with the average of three readings utilized for analysis.

### Measurement of baseline information

The investigators collected baseline data from participants through questionnaire surveys, which included demographic characteristics (such as including age, gender, and ethnic group), lifestyle (such as tobacco smoking, alcohol consumption, and household income), geographical locations (urban or rural), and other variables such as occupation and educational attainment. The questionnaires utilized in the study was developed by the Chinese Center for Disease Control and Prevention and can be found in the Supplementary documents.

### Biochemical measurements

Blood samples were obtained from participants following an overnight fast of a minimum of 8 h, and were analyzed for levels of total cholesterol (TC), triglycerides (TG), high-density lipoprotein cholesterol (HDL-C), and low-density lipoprotein cholesterol (LDL-C) according to standard protocols. Lipid abnormalities were categorized based on the parameters outlined in the 2016 Chinese guidelines for the management of dyslipidemia in adults [25].

### Comprehensive measurement of SES

This study utilized the scoring method to assess indicators of household income, personal education level, and occupation in accordance with the classification of socio-economic status (SES) by both domestic and foreign scholars, including Torres et al. [26] and Lei et al. [27]. The scoring system assigned values ranging from 1 to 6 points, representing low to high ranks for each indicator, and the total score was calculated based on these values. Subsequently, the SES was categorized into distinct levels based on the total score. The criteria for scoring measures of socio-economic status are outlined in Table 1.

**Table 1:** Scoring criteria for measures of socio-economic status

Income	Education	Occupation	Scores
≥ 13500	College	Managerial and administrative	6
8000–13500	Associate degree	Professional specialty	5
5200–8000	Senior high school	Sales and service	4
3600–5200	Junior high school	Precision production, farming/ forestry/fishing	3
2000–3600	Elementary school	Other laborers	2
< 2000	Illiteracy	Unemployed person	1

### Diagnostic criteria and relevant definitions

(1) The diagnostic criteria for abnormal blood pressure encompassed a physician's assessment of abnormal blood pressure, utilization of blood pressure medications, or meeting the criteria of systolic blood pressure ≥ 140 mmHg and/or diastolic blood pressure ≥ 90 mmHg [28]. (2) The diagnostic criteria for dyslipidemia involved a physician's diagnosis of dyslipidemia, use of lipid-lowering medications, total cholesterol (TC) levels ≥ 6.22 mmol/L, triglycerides (TG) levels ≥ 2.26 mmol/L, high-density lipoprotein cholesterol (HDL-C) levels < 1.04 mmol/L, or low-density lipoprotein cholesterol (LDL-C) levels ≥ 4.14 mmol/L [29, 30]. (3) Alcohol consumption is defined as consuming alcohol within the past month and consuming alcohol at least once per week [31, 32]. (4) Insufficient sleep is characterized by a duration of less than 7 h per day [33, 34]. (5) Insufficient fruit intake is defined as consuming less than 200 g of fruit per day, insufficient vegetable intake is consuming less than 300 g of vegetables per day [35, 36], and excessive salt intake is consuming 6 g or more of salt per day [37].

### Statistical analysis

Categorical variables were characterized by their frequency and percentage, while continuous variables were summarized by their mean and standard deviation. The Epidata 3.1 software was employed for database creation and data entry, with statistical analyses performed using SPSS 20.0 software. The chi-square test was employed to assess the statistical differences among categorical variables t, while the Cox proportional hazards regression model was applied to evaluate the association between SES and the incidence of hypertension. The Forest plot package within the R 4.0.3 software was utilized to generate a forest plot illustrating the relationship between socioeconomic status and the risk of hypertension. All statistical tests conducted were two-sided, with a significance level of *P* < 0.05 deemed as statistically significant.

## Results

### Baseline characteristics

A total of 3546 participants were enrolled for the analysis after excluding 5734 people who met the exclusion criteria. The median follow-up duration was 6.43 years. Among the participants, 1924 (54.3%) were female, 2195 (61.9%) were of Han nationality, and 2428 (68.5%) resided in rural areas. Factors such as age, marital status, educational level, household income, occupation, cigarette smoking, alcohol consumption, and excessive salt intake were found to be significantly associated with the incidence of hypertension among the participants. Further details can be found in Table 2.

**Table 2:** Basic characteristics

Characteristics	Total (%)	Hypertension (%)	Non-Hypertension (%)	Χ^2^ -value	*P*-value
**Age (years)**				100.785	< 0.001
18–45	2159 (60.9)	375 (17.4)	1784 (82.6)		
45–60	1025 (28.9)	280 (27.3)	745 (72.7)		
≥ 60	362 (10.2)	140 (38.7)	222 (61.3)		
**Gender**				3.875	0.052
Male	1622 (45.7)	388 (23.9)	1234 (76.1)		
Female	1924 (54.3)	407 (21.2)	1517 (78.8)		
**Ethic Group**				0.326	0.568
Han nationality	2195 (61.9)	499 (22.7)	1696 (77.3)		
Ethnic minorities	1351 (38.1)	296 (21.9)	1055 (78.1)		
**Marital status**				17.390	< 0.001
Unmarried	332 (9.4)	45 (13.6)	287 (86.4)		
Married/Cohabitation	2907 (82.0)	672 (23.1)	2235 (76.9)		
Divorce/ Separation	307 (8.7)	78 (25.4)	229 (74.6)		
**Location**				7.056	0.008
Urban	1118 (31.5)	220 (19.7)	898 (80.3)		
Rural	2428 (68.5)	575 (23.7)	1853 (76.3)		
**Income (yuan)**^**a**^				15.802	0.007
< 2000	490 (13.9)	129 (26.3)	361 (73.7)		
2000–3600	682 (19.4)	158 (23.2)	524 (76.8)		
3600–5200	586 (16.6)	133 (22.7)	453 (77.3)		
5200–8000	524 (14.9)	120 (22.9)	404 (77.1)		
8000–13500	648 (18.4)	149 (23.0)	499 (77.0)		
≥ 13500	592 (16.8)	99 (16.7)	493 (83.3)		
**Educational status**				46.042	< 0.001
Illiteracy	528 (14.9)	171 (32.4)	357 (67.6)		
Elementary school	1265 (35.7)	297 (23.5)	968 (76.5)		
Junior high school	1158 (32.7)	223 (19.3)	935 (80.7)		
Senior high school	407 (11.5)	71 (71.4)	336 (82.6)		
Associate degree	133 (3.8)	24 (18.0)	109 (82.0)		
College	55 (1.6)	9 (16.4)	46 (83.6)		
**Occupation**				14.416	0.013
Unemployed person	511 (14.4)	114 (22.3)	397 (77.7)		
Other laborers	323 (9.1)	63 (19.5)	260 (80.5)		
Precision production, farming/ forestry/fishing	2190 (61.8)	529 (24.2)	1661 (75.8)		
Sales and service	182 (5.1)	30 (16.5)	152 (83.5)		
Professional specialty	286 (8.1)	48 (16.8)	238 (83.2)		
Managerial and administrative	54 (1.5)	11 (20.4)	43 (79.6)		
**Cigarette smoking **^**a**^				9.878	0.007
Current smoker	975 (28.6)	249 (25.5)	726 (74.5)		
Former smoker	123 (3.6)	36 (29.3)	87 (70.7)		
Never smoker	2306 (67.7)	492 (21.3)	1814 (78.7)		
**Alcohol use**				8.467	0.004
No	2932 (82.7)	630 (21.5)	2302 (78.5)		
Yes	614 (17.3)	165 (26.9)	449 (73.1)		
**BMI (kg/m**^**2**^**)**				9.969	0.019
< 18.5	218 (6.2)	38 (17.4)	180 (82.6)		
18.5–24.0	2311 (65.3)	502 (21.7)	1809 (78.3)		
24.0–28.0	841 (23.8)	204 (24.3)	637 (75.7)		
≥ 28.0	171 (4.8)	50 (29.2)	121 (70.8)		
**Insufficient fruit intake **^**a**^				0.952	0.329
No	2394 (67.8)	525 (21.9)	1869 (78.1)		
Yes	1137 (32.2)	266 (23.4)	871 (76.6)		
**Insufficient vegetable intake **^**a**^				5.181	0.023
No	247 (7.0)	41 (16.6)	206 (83.4)		
Yes	3276 (93.0)	749 (22.9)	2527 (77.1)		
**Insufficient sleep**				4.247	0.039
No	3104 (87.5)	679 (21.9)	2425 (78.1)		
Yes	442 (12.5)	116 (26.2)	326 (73.8)		
**Excessive salt intake**				4.007	0.045
No	1010 (28.5)	204 (20.2)	806 (79.8)		
Yes	2536 (71.5)	591 (23.3)	1945 (76.7)		
**Dyslipidemia**				1.679	0.195
No	1309 (36.9)	309 (23.6)	1000 (76.4)		
Yes	2237 (36.1)	486 (21.7)	1751 (78.3)		

^a^ the variable has a missing value, *BMI* body mass index

### Multivariate analysis of SES single index and hypertension incidence

**Fig. 2:**
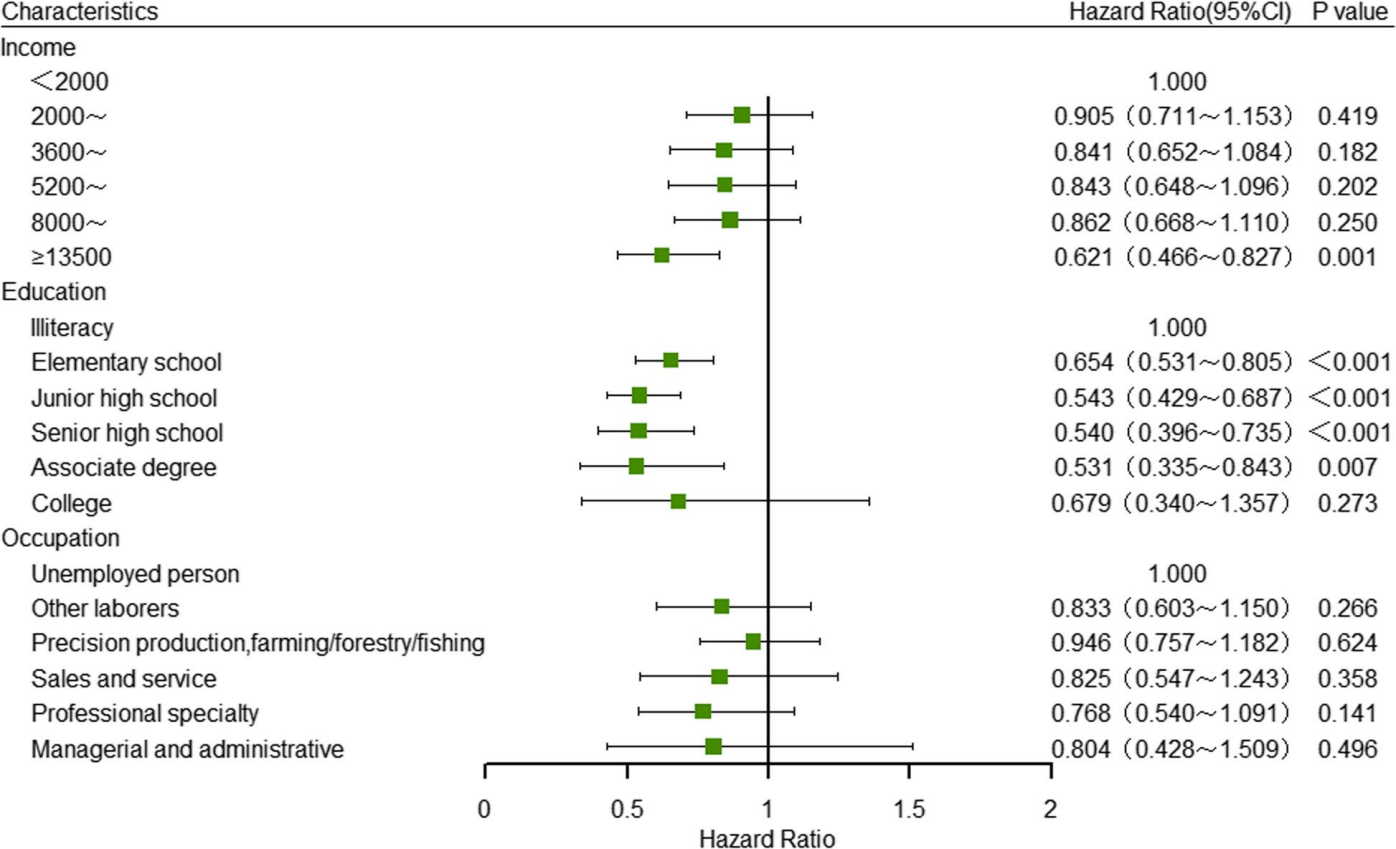
Cox regression analysis of single index of SES on the incidence of hypertension

In the Cox proportional hazards regression model for multivariate analysis, household income, educational level and occupation were considered as independent variables with the incidence of hypertension as the dependent variable (Table 3). The results indicated that, after controlling for confounding factors, adults in the highest household income group had a significantly lower risk of hypertension compared to those in the lowest income group (HR = 0.636, 95% CI: 0.478–0.845). No significant differences were observed in the risk of hypertension among other income groups when compared to the lowest income group. Furthermore, in terms of educational attainment, individuals who graduated from primary school, junior high school, high school and associate degree programs exhibited respectively decreases in the risk of hypertension compared to the illiterate population: 34.6% (HR = 0.654, 95%CI: 0.53–0.805), 45.7% (HR = 0.543, 95%CI: 0.429–0.687), 46.0% (HR = 0.540, 95%CI: 0.396–0.735) and 46.9% (HR = 0.531, 95%CI: 0.335–0.843). Interestingly, no significant difference was observed in the incidence of hypertension based on occupation (*P* > 0.05) (Fig. 2).

**Table 3:** Multivariate Cox regression analysis of SES single index on the incidence of hypertension

Characteristics	Hypertension	Incidence density^a^	HR(95%CI)	
			Model1	Model2
**Income**				
< 2000	129	39.13	1.000	1.000
2000–3600	158	34.27	0.912(0.720–1.154)	0.905(0.711–1.153)
3600–5200	133	33.20	0.828(0.646–1.060)	0.841(0.652–1.084)
5200–8000	120	32.36	0.807(0.624–1.044)	0.843(0.648–1.096)
8000–13500	149	32.63	0.815(0.636–1.046)	0.862(0.668–1.110)
≥ 13500	99	23.78	0.591(0.447–0.782) ^***^	0.621(0.466–0.827)^**^
**Education**				
Illiteracy	171	50.66	1.000	1.000
Elementary school	297	34.21	0.639(0.522–0.783) ^***^	0.654(0.531–0.805)^***^
Junior high school	223	26.95	0.502(0.400–0.631) ^***^	0.543(0.429–0.687)^***^
Senior high school	71	24.76	0.492(0.364–0.665) ^***^	0.540(0.396–0.735)^***^
Associate degree	24	25.68	0.509(0.325–0.797) ^**^	0.531(0.335–0.843)^**^
College	9	23.92	0.523(0.264–1.038)	0.679(0.340–1.357)
**Occupation**				
Unemployed	114	32.15	1.000	1.000
Other laborers	63	29.36	0.964(0.703–1.322)	0.833(0.603–1.150)
Precision production, farming/ forestry/fishing	529	34.97	1.027(0.824–1.279)	0.946(0.757–1.182)
Sales and service	30	22.64	0.773(0.514–1.164)	0.825(0.547–1.243)
Professional specialty	48	24.11	0.818(0.579–1.156)	0.768(0.540–1.091)
Managerial and administrative	11	29.50	0.862(0.460–1.613)	0.804(0.428–1.509)

*HR* hazard ratio; 95% CI: 95% confidence interval

^***^P < 0.001

^**^P < 0.01

^*^P < 0.05

^a^Per 1000 person-years. Model 1 was adjusted for age, sex, ethnicity, marital status, urban and rural residence and BMI classification; On the basis of Model 1, Model 2 were further adjusted for variables including smoking, drinking, insufficient intake of fresh vegetables and fruits, insufficient sleep, excessive salt intake, and dyslipidemia

### Univariate analysis of the comprehensive score of SES and hypertension incidence

The individual indicators of socioeconomic status (SES), including household income, individual education level, and occupation, were ranked from lowest to highest on a score of 1 to 6. Subsequently, the total SES score was calculated by summing these individual scores. Finally, the socioeconomic status was categorized into 8 distinct levels based on the eighth digit of the comprehensive score. Through univariate Cox regression analysis, it was determined that individuals with scores of 7, 8, 9, 10, 11, 12, and greater than 12 had a decreased risk of hypertension compared to those with a score of 6 or less. The risk reduction percentages were as follows: 31.0% (HR = 0.690, 95%CI: 0.546–0.872), 34.5% (HR = 0.655, 95%CI: 0.519–0.826), 33.3% (HR = 0.667, 95%CI: 0.528–0.843), 46.7% (HR = 0.533, 95%CI: 0.414–0.687), 50.0% (HR = 0.500, 95%CI: 0.379–0.660), 51.2% (HR = 0.488, 95%CI: 0.336–0.707), and 58.8% (HR = 0.412, 95%CI: 0.308–0.552) respectively (Table 4).

**Table 4:** Univariate Cox regression analysis of SES comprehensive score on the incidence of hypertension

SES comprehensive score	Hypertension, n	Incidence density^a^	HR(95% CI)
≤ 6	183	42.64	1.000
7	115	32.91	0.690(0.546–0.872) ^**^
8	119	32.78	0.655(0.519–0.826) ^***^
9	115	34.27	0.667(0.528–0.843) ^**^
10	92	29.62	0.533(0.414–0.687) ^***^
11	71	29.34	0.500(0.379–0.660) ^***^
12	33	26.61	0.488(0.336–0.707) ^***^
> 12	60	21.32	0.412(0.308–0.552) ^***^

*HR* hazard ratio, *95% CI* 95% confidence interval

^***^P < 0.001

^**^P < 0.01

^*^P < 0.05

^a^Per 1000 person-years

### Multivariate analysis of the comprehensive SES score and the incidence of hypertension.

**Fig. 3:**
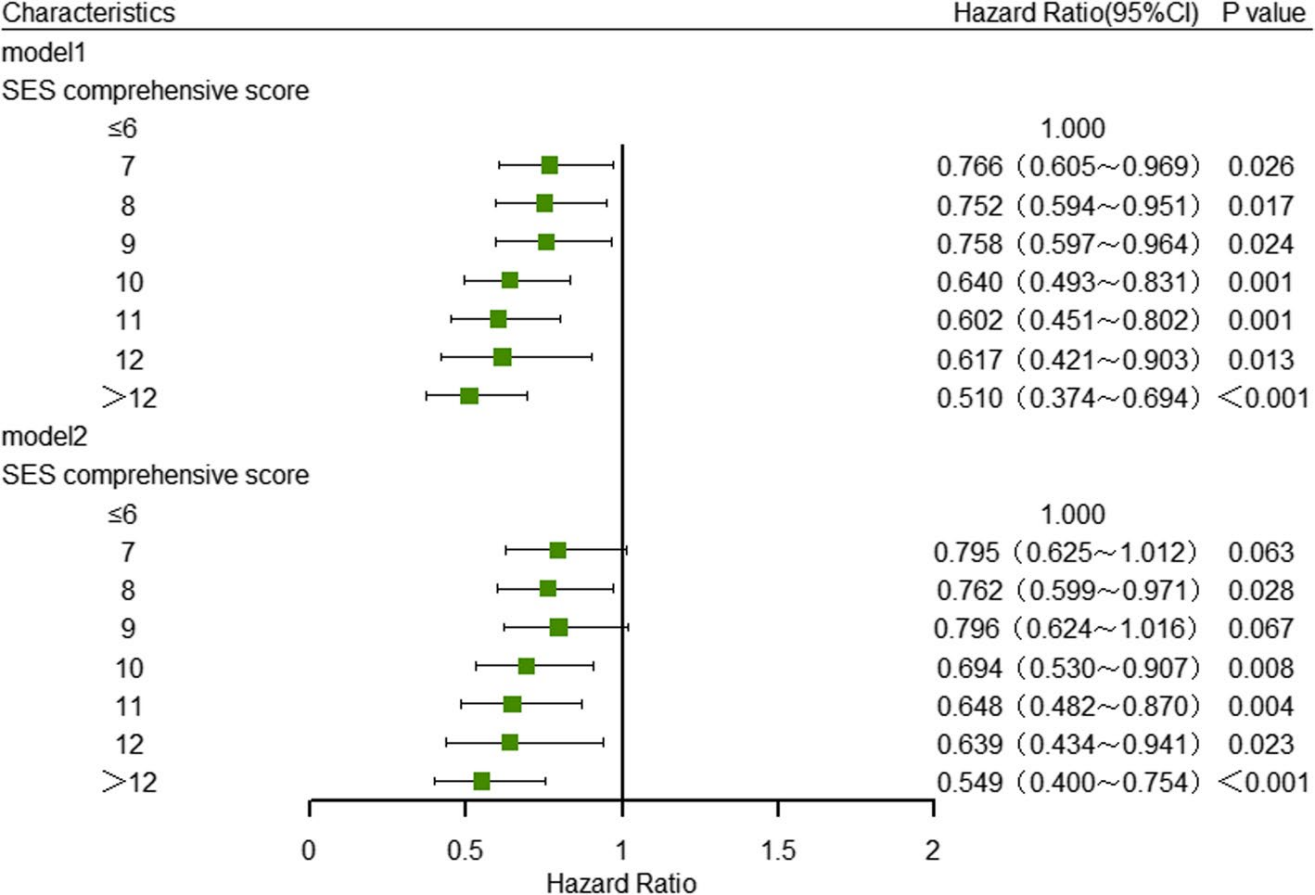
Cox regression analysis of comprehensive score of SES on hypertension incidence

Individuals with varying levels of socioeconomic status demonstrated differing rates of hypertension. Upon controlling for all potential confounding variables, it was observed that compared with individuals with a score of 6 or lower, the risk of hypertension in participants with scores of 8, 10, 11, 12, and greater than 12 decreased respectively by 23.9% (HR = 0.761, 95%CI: 0.598–0.969), 29.7% (HR = 0.703, 95%CI: 0.538–0.919), 34.0% (HR = 0.660, 95%CI: 0.492–0.885), 34.3% (HR = 0.657, 95%CI: 0.447–0.967), 43.9% (HR = 0.561, 95%CI: 0.409–0.769). Nonetheless, there was no statistically significant variance in the likelihood of developing hypertension between the group with a score of 6 or lower 6 and the group with a score of 9 (Table 5) (Fig. 3**)**.

**Table 5:** Multivariate Cox regression analysis of SES comprehensive score on hypertension incidence

SES scores	Hypertension	Incidence density^a^	HR(95%CI)	
			Model1	Model2
≤ 6	183	42.64	1.000	1.000
7	115	32.91	0.766(0.605–0.969) ^*^	0.795(0.625–1.012)
8	119	32.78	0.752(0.594–0.951) ^*^	0.762(0.599–0.971) ^*^
9	115	34.27	0.758(0.597–0.964) ^*^	0.796(0.624–1.016)
10	92	29.62	0.640(0.493–0.831) ^**^	0.694(0.530–0.907) ^**^
11	71	29.34	0.602(0.451–0.802) ^**^	0.648(0.482–0.870) ^**^
12	33	26.61	0.617(0.421–0.903) ^*^	0.639(0.434–0.941) ^*^
> 12	60	21.32	0.510(0.374–0.694) ^***^	0.549(0.400–0.754) ^***^

*HR* hazard ratio, *95% CI* 95% confidence interval

^***^P < 0.001

^**^P < 0.01

^*^P < 0.05

^a^Per 1000 person-years. Model 1 was adjusted for age, sex, ethnicity, marital status, urban and rural residence and BMI classification; On the basis of Model 1, Model 2 were further adjusted for variables including smoking, drinking, insufficient intake of fresh vegetables and fruits, insufficient sleep, excessive salt intake, and dyslipidemia

## Discussion

Various researchers have presented differing conclusions on the relationship between individual income and the prevalence of hypertension. Harshfield et al. [38] and Glover et al. [39] observed a positive association between individual income and hypertension among African American adults, while Rosengren et al. [40] suggested that higher income was associated with a reduced risk of hypertension. However, Conen et al. [41] found that no significant relationship between individual income and the incidence of hypertension. In our research, it was observed that individuals in the highest household income bracket (≥ 13500 yuan) had a 0.621 times lower risk of hypertension compared to those in the lowest income bracket (< 2000 yuan). The risk of hypertension in other income levels did not show significant differences when compared to the lowest income. By analyzing the reasons, the lack of precise income information provided by participants could potentially impact the accuracy of the data analysis [42]. Additionally, household income is a variable that is susceptible to external influences and misclassification errors [43].

The educational background of individuals has been shown to influence the incidence of hypertension. Vathesatogkit et al. [43] and Mosca et al. [44] demonstrated that individuals with a higher levels of education have a reduced risk of hypertension. Consistent with these findings, our study also found that individuals with varying levels of education, from primary school to junior college, had significantly lower risks of hypertension compared to the illiterate population. Specifically, the risk of hypertension decreased respectively by 34.4%, 44.9%, 44.9% and 46.1% for those who graduated from primary school, junior high school, senior high school, and junior college. Collectively, these results indicated that an individual’s level of education significantly influences the incidence of hypertension, with higher education levels correlating with lower risk. Study by Vallée et al. [45] also came to the similar conclusion. This relationship may be explained by the increased awareness and knowledge individuals gain through education, leading to better understanding of hypertension risk factors, preventive measures, and healthier lifestyle choices, ultimately reducing the likelihood of developing hypertension [42].

In the realm of occupational influences on the incidence of hypertension, research conducted by Bhattarai et al. [46] demonstrated a heightened risk of hypertension among employed individuals compared to their unemployed counterparts. Demos et al. [47] observed that farmers exhibited a lower likelihood of hypertensin compared to individuals in alternative occupations (businessmen, construction workers, craftsmen, etc.). Additionally, Davis-Lameloise et al. [48] highlighted a higher prevalence of hypertension among adults engaging in higher level of physical activity. Contrary to these findings, our study revealed no significant variance in hypertension risk across different occupational groups.

Utilizing individual socioeconomic status (SES) indicators, such as household income, educational attainment, and occupation, to investigate the association with hypertension may introduce potential bias [49]. It is imperative to consider multiple indicators of SES when examining the relationship between SES and hypertension, as the influence of SES on hypertension may be the result of multiple factors such as family income, personal education level and occupation. Numerous studies have demonstrated a negative correlation between SES and hypertension in high-income countries [18, 50]. However, findings regarding the relationship between socioeconomic status (SES) and hypertension prevalence vary among low- and middle-income countries. For instance, a study conducted in rural Mexico [51] demonstrated a positive correlation between economic status and hypertension prevalence, while Gulliford et al. [22] found no significant association between SES and hypertension prevalence. However, our study revealed a gradual decrease in the risk of hypertension with improving SES. This is consistent with the findings of a study by Kirschbaum et al. [52], which also identified SES as a protective factor against hypertension. There are multiple reasons contributing to this outcome. Firstly, individuals with a higher SES exhibit a greater propensity towards adopting a healthy lifestyle due to their elevated levels of education, household income, and heightened health consciousness, which enables them to access a greater wealth of information regarding hypertension [40]. In addition, individuals with a higher SES have increased access to medical and health-related resources, and demonstrate a greater willingness and capacity to invest time and effort in prioritizing their health. Consequently, enhancing socioeconomic status has the potential to mitigate the incidence of hypertension.

In conclusion, the findings suggest a negative correlation between socioeconomic status and hypertension incidence among the population in Southwest China, indicating that individuals with lower SES are at a higher risk of developing hypertension. Therefore, efforts to prevent and control hypertension in this region should prioritize interventions targeting those with lower SES, who are more likely to have lower household income, lower education level and engage in manual labor.

Given that the survey participants were adult residents in Southwest China, who are generally difficult to change their educational level, it is imperative to concentrate on modifying residents' household income and occupation. This may include enhancing employment opportunities and augmenting personal income for residents. In addition, it is necessary for both society and the public media to disseminate information and provide education on the prevention and management of hypertension This will serve to enhance individuals' health awareness and literacy, promote the adoption of healthy behaviors and lifestyles, and ultimately decrease the prevalence of hypertension within the population [52].

## Conclusion

In conclusion, enhancing socioeconomic status has been shown to lower the incidence of hypertension, with a negative correlation observed between socioeconomic status and hypertension incidence among adults in southwest China. Therefore, a higher socioeconomic status serves as a protective factor against the development of hypertension in this population.

### Supplementary Information

**Supplementary Material 1. **

## Data Availability

The datasets generated and/or analyzed during the current study are not publicly available due to the risk of compromising individual privacy but are available from the corresponding author on reasonable request and provided that an appropriate collaboration agreement can be agreed upon.
