# Supplementary material for: Relationship between socioeconomic status and hypertension incidence among adults in southwest China: a population-based cohort study
Source: BMC Public Health. 2024 May 2;24:1211. doi: 10.1186/s12889-024-18686-5 (PMC11064324; doi:10.1186/s12889-024-18686-5)
Supplement: Supplementary file 1 — Supplementary Material 1. [file 12889_2024_18686_MOESM1_ESM.zip › Baseline Questionnaire - Family Part.docx]

| Family code |
| --- |

The central government transfers payments to local governments

**Chronic Disease Surveillance in China (2010)**

**Family questionnaire**

| Name：________________ Telephone number：______________________ | | |
| --- | --- | --- |
| Name of monitoring site： | Monitoring point code： 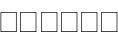 | |
| Name of town/street： | Town/street code： | 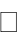 |
| Name of village/neighborhood committee： | Village/neighborhood committee code： | 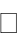 |
| Whether it is a family household after replacement  1 Yes  2 No | Family code： 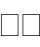 | |
| Signature of investigator： ________________  Date： 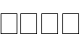 year 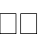 month 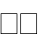 day | Signature of monitoring point quality controller： ______________________  Date： 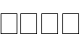 year 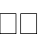 month 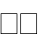 day | |
| Signature of provincial quality controller： _______________________  Date： 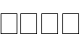 year 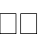 month 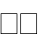 day | | |

Chinese Center for Disease Control and Prevention

Center for Prevention and Control of Chronic Non communicable Diseases

August 2010

| Family Registration Form |
| --- |

Fill in the information of a family member who meets the conditions of permanent residence in each row of the following family member registration form. Fill in according to the age of the first male and then female.

Refer to the HH4 age in the family registration form. If the age is greater than or equal to 18 years old, fill in 1 in HH5. If the age is less than 18 years old, fill in 2 in HH5.

The family members whose HH5 is filled in with 1 are numbered in HH6 in descending order of age from male to female.

| Name  **(HH1)** | Relationship code with the head of household  **(HH2)** | Gender  **1=**Male **2=**Female  **(HH3)** | Age  **(HH4)** | Whether the age is 18 or above  **1=Yes 2=**No  **(HH5)** | Family member number **(HH6)** |
| --- | --- | --- | --- | --- | --- |
|  |  |  |  |  |  |
|  |  |  |  |  |  |
|  |  |  |  |  |  |
|  |  |  |  |  |  |
|  |  |  |  |  |  |
|  |  |  |  |  |  |
|  |  |  |  |  |  |
|  |  |  |  |  |  |
|  |  |  |  |  |  |
|  |  |  |  |  |  |

**Note:** The relationship code with the head of household: 1=head of household, 2=spouse, 3=son or daughter, 4=daughter-in-law or son-in-law, 5=grandchild or grandchild, 6=parents, 7=parents in law, 8=brothers or sisters, 9=grandparents, 10=other relatives, 11=no kinship (friends, servants, boarders, boarders, others)

Query the KISH form assigned to the family, and select the family members to receive the personal questionnaire survey.

| HH7 | KISH table assigned to the family  A 、B1 、B2 、C 、D 、E1 、E2 、F | 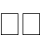 |
| --- | --- | --- |
| HH8 | Number of selected family members | 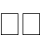 |

| Household diet and fuel use |
| --- |

| Family diet condition | | | |
| --- | --- | --- | --- |
| HH9a | How many people usually have breakfast at home? | 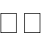 people | |
| HH9b | How many people in your family usually have lunch at home? | 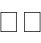 people | |
| HH9c | How many people in your family usually have dinner at home? | 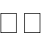 people | |
| HH10 | How much vegetable oil do you usually eat in a month?  **Note to investigators**: If you do not know or remember clearly, fill in "- 9" on the right before the decimal point, the same below. | 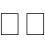 . 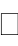 500g | |
| HH11 | How much animal oil do you usually eat in a month? | 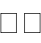 . 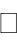 500g | |
| HH12 | How much salt do you usually eat in a month? | 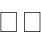 . 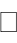 500g | |
| HH13 | How much soy sauce do you usually eat in a month? | 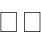 . 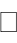 500g | |
| HH14 | How much sugar do you usually eat in a month? | 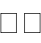 . 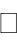 500g | |
| HH15 | How many pickles do you usually eat in your family? | 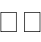 . 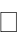 500g | |
| HH16 | How many pickles do you usually eat in your family each month? | 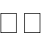 . 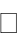 500g | |
| HH17 | How much fermented bean curd do you usually eat in your family? | 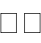 . 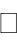 500g | |
| HH18 | How much sauce do you usually eat in a month? | 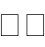 . 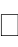 500g | |
| HH19a | Have you received any salt spoons at home? | 1 Yes  2 No …………………… to  99 Unclear…………………… to | HH20a  HH20a |
| HH19b | If so, where did you get it? | 1 Issued by neighborhood committee  2 Buy it yourself  3 Gift from relatives and friends  88 Other | |
| HH20a | Have you ever received an oil control pot at home? | 1 Yes  2 No ……………………… to  99 Unclear …………………… to | HH21  HH21 |

| HH20b | If so, where did you get it? | 1 Issued by neighborhood committee  2 Buy it yourself  3 Gift from relatives and friends  88 Other |
| --- | --- | --- |
| HH21 | Who is mainly responsible for purchasing food in your family (fill in the family member number)?  **Note to the investigato**r: if you do not know, fill in "- 9"; If the person responsible for purchasing food does not have a family member number, fill in "88". | 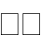 |
| Fuel usage | | |
| HH22 | What is the main fuel your family uses for cooking? | 1 Firewood/charcoal/wood/animal excrement  2 Coal  3 Gas/liquefied gas/natural gas/biogas  4 Solar energy/electricity  88 Other |
| HH23 | Do you use a range hood or an exhaust fan when cooking in your home? | 1 Yes  2 No |
| HH24 | Where do you cook at home? | 1 Living room or bedroom  2 Separate kitchen  3 Outdoor  88 Other： |

| Family economic status |
| --- |

| HH25 | What is your family's total income in 2009?  **Note to the investigator:** only one of the annual income and monthly income is recorded。 | 1 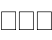, 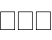yuan/month  **or**  2 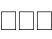, 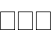yuan/month  99 Don't know the specific income  97 Refuse to answer |
| --- | --- | --- |
| HH26 | What is your family's total expenditure in 2009?  **Note to the investigator**: only one of the annual and monthly expenditures is recorded. | 1 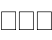, 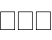yuan/month  **or**  2 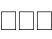, 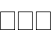yuan/month  99 Don't know the specific income  97 Refuse to answer |

| **Health problems of people aged 50 and above** |
| --- |

| The following questions should be asked of family members ≥ 50 years old  If there is no member ≥ 50 years old ... ………………………………………… ... **to** | | | | | End of questionnaire |
| --- | --- | --- | --- | --- | --- |
| Note to the investigator: Every member of the family ≥ 50 years old must be interviewed. If there are more than 4 family members who meet the requirements, 4 older members are preferred to fill in. | | Family member number | | | |
|  |  | 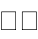 | 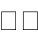 | 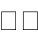 | 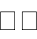 |
| HH27 | What is your education level?  1 Not receiving formal school education  2 Not graduated from primary school  3 Graduated from primary school  4 Junior high school graduation  5 High school/technical school  6 College Graduation  7 Bachelor degree  8 Postgraduate or above | 1  2  3  4  5  6  7  8 | 1  2  3  4  5  6  7  8 | 1  2  3  4  5  6  7  8 | 1  2  3  4  5  6  7  8 |
| HH28 | Your current marital status?  1 Unmarried  2 Married  3 Cohabitation  4 Widow  5 Divorce  6 Separation  88 Other | 1  2  3  4  5  6  88 | 1  2  3  4  5  6  88 | 1  2  3  4  5  6  88 | 1  2  3  4  5  6  88 |
| HH29 | What is your occupation?  1 Agriculture, forestry, animal husbandry, fishery and water conservancy  2 Production and equipment operators and relevant personnel  3 Business and service personnel  4 Person in charge of state organs, party mass organizations, enterprises and public institutions  5 Office staff and relevant personnel  6 Professional technicians  7 Soldier  8 Other workers  9 Students  10 Unemployed  11 Housework  12 Retired personnel | 1  2  3  4  5  6  7  8  9  10  11  12 | 1  2  3  4  5  6  7  8  9  10  11  12 | 1  2  3  4  5  6  7  8  9  10  11  12 | 1  2  3  4  5  6  7  8  9  10  11  12 |

| HH30 | What kind of medical insurance do you currently have? (**Multiple choices are allowed**)  Note to investigator: answer must be read  1 Basic medical insurance for urban employees  2 Free medical treatment  3 Medical insurance for urban residents  4 New rural cooperative medical care  5 Commercial medical insurance  6 Other  7 Didn't participate  99 Unclear | 1  2  3  4  5  6  7  99 | 1  2  3  4  5  6  7  99 | 1  2  3  4  5  6  7  99 | 1  2  3  4  5  6  7  99 |
| --- | --- | --- | --- | --- | --- |
| HH31 | Have you had trouble sleeping in the past 12 months?  1 Often  2 Occasionally  3 No | 1  2  3 | 1  2  3 | 1  2  3 | 1  2  3 |
| HH32 | Does your sense of smell change?  1 Yes  2 No | 1  2 | 1  2 | 1  2 | 1  2 |
| HH33 | Do you have any problems with constipation?  1 Often  2 Occasionally  3 No | 1  2  3 | 1  2  3 | 1  2  3 | 1  2  3 |
| HH34 | Do you think your memory is worse than that of relatives, neighbors or colleagues of the same age?  1 Yes  2 No | 1  2 | 1  2 | 1  2 | 1  2 |
| HH35 | Do your family members, neighbors or colleagues think your memory is poor?  1 Yes  2 No | 1  2 | 1  2 | 1  2 | 1  2 |
| HH36 | Is your memory much lower than it was a year ago?  1 Yes  2 No | 1  2 | 1  2 | 1  2 | 1  2 |
| If the above three questions HH34-HH36 are answered "No" **……….…...…** to | | | | | **HH41** |
| HH37 | Do you need long-term care in your daily life due to poor memory?  1 Yes  2 No | 1  2 | 1  2 | 1  2 | 1  2 |
|  | If HH37 selects "2 Not Required", then …………………………… to | | | | **HH39** |

| HH38 | **Who took care of you?**  1 Family  2 Nanny  3 Professional nursing staff  4 Nobody cares | 1  2  3  4 | | | | | | 1  2  3  4 | | | | 1  2  3  4 | | | | | | 1  2  3  4 | | | | |
| --- | --- | --- | --- | --- | --- | --- | --- | --- | --- | --- | --- | --- | --- | --- | --- | --- | --- | --- | --- | --- | --- | --- |
| HH39 | Have you ever been to the hospital because of memory loss?  1 Yes  2 No | 1  2 | | | | | | 1  2 | | | | 1  2 | | | | | | 1  2 | | | | |
| HH40 | Are you diagnosed with senile dementia in hospitals above county/district level?  1 Yes  2 No | 1  2 | | | | | | 1  2 | | | | 1  2 | | | | | | 1  2 | | | | |
| HH41 | Have you ever heard of Parkinson's disease or Parkinson's syndrome?  1 Yes  2 No | 1  2 | | | | | | 1  2 | | | | 1  2 | | | | | | 1  2 | | | | |
| HH42 | Have you experienced any of the following symptoms in the past few years?  (**Multiple choices are allowed**)  1Both arms tremble or tremble involuntarily  2 Shaking or trembling of legs, feet or chin  3 The body cannot be straightened, the body bends forward when standing or walking, the sense of balance is poor, and it is easy to fall  4 Slow movement, dragging feet or taking small steps  5 Speak slowly with a low intonation  6 Write smaller and smaller  7 No symptoms above | 1  2  3  4  5  6  7 | | | | | | 1  2  3  4  5  6  7 | | | | 1  2  3  4  5  6  7 | | | | | | 1  2  3  4  5  6  7 | | | | |
|  | If HH42 selects "7 without any of the above symptoms", then**………to** | | | | | | | | | | | | | | | | | **HH50** | | | | |
| HH43 | When did the symptoms begin to appear?  **Note to the investigator**: only one item1 Which yea?  Or  2 At what age |  | | | | | |  | | | |  | | | | | |  | | | | |
|  |  |  | |  |  | |  |  |  |  |  |  |  | |  | |  |  |  | |  |  |
|  |  |  | | | |  | |  | | |  |  | | | |  | |  | | | |  |
|  |  |  |  | |  |  |  |  |  |  |  |  |  |  | |  |  |  |  |  | |  |
| HH44 | Does the symptom now occur every day?  1 Yes  2 No  3 The symptoms are gone | 1  2  3 | | | | | | 1  2  3 | | | | 1  2  3 | | | | | | 1  2  3 | | | | |
| HH45 | Are the symptoms getting worse?  1 Yes  2 No | 1  2 | | | | | | 1  2 | | | | 1  2 | | | | | | 1  2 | | | | |

| HH46 | Did you go to the hospital to see a doctor because of the above symptoms?  1 Yes  2 No | 1  2 | 1  2 | 1  2 | 1  2 |
| --- | --- | --- | --- | --- | --- |
| HH47 | Have you ever been diagnosed with Parkinson's disease or Parkinson's syndrome in hospitals above county/district level?  1 Yes  2 No | 1  2 | 1  2 | 1  2 | 1  2 |
| HH48 | Do you need long-term care for your daily life because of the above physical symptoms that cause mobility inconvenience?  1 Yes  2 No | 1  2 | 1  2 | 1  2 | 1  2 |
|  | If HH48 selects "2 Not Required", then……………………………to | | | | HH50 |
| HH49 | Who took care of you?  1 Family  2 Nanny  3 Professional nursing staff  4 Nobody cares | 1  2  3  4 | 1  2  3  4 | 1  2  3  4 | 1  2  3  4 |
| HH50 | Who answered the above questions about old age?  **Note to the investigato**r: please write down the family member number of the respondent or substitute. | 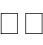 | 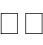 | 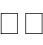 | 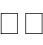 |
