# Supplementary material for: Relationship between socioeconomic status and hypertension incidence among adults in southwest China: a population-based cohort study
Source: BMC Public Health. 2024 May 2;24:1211. doi: 10.1186/s12889-024-18686-5 (PMC11064324; doi:10.1186/s12889-024-18686-5)
Supplement: Supplementary file 1 — Supplementary Material 1. [file 12889_2024_18686_MOESM1_ESM.zip › Baseline Questionnaire - Personal Part.docx]

| **Where you stick your personal code.**   \| With or without diabetes: ____ \| \| --- \|   **CNCDS2010-IQ** |
| --- | --- |

The central government transfers payments to local governments

**Chronic Disease Surveillance in China (2010)**

**Personal questionnaire**

| Name of respondents: __________________ Telephone: _____________________ | | | |
| --- | --- | --- | --- |
| Name of Monitoring site (County/District): | Monitoring point Code: 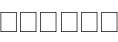 | | |
| Name of town/Street: | Township/Street Number: | | 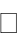 |
| Name of Village/Neighborhood  Committee: | Village/Neighborhood Committee No. | | 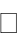 |
| Family Code: 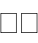 | | | |
| Signature of Investigator: _____________  Date：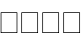 . 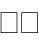 .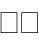 | | Signature of quality controller： ______________  Date：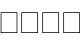. 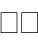 . 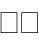 | |
| Signature of provincial quality controller： _______________________  Date：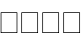 . 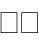 .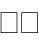 | | | |

Chinese Center for Disease Control and Prevention

Center for the Prevention and Control of Chronic Noncommunicable Diseases

August 2010

| Attach the blood collection code strip |
| --- |

Survey starting time (24 hours):
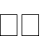
 hour
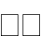
 minutes

| **Part one Basic information** | | | | | |
| --- | --- | --- | --- | --- | --- |
| A1 | Your birthdate | 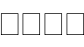 year 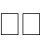 month 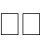 day | | | |
| A2 | Your gender | 1 Male  2 Female | | | |
| A3 | Your nation | 1  2  3  4  5  6 | Han nationality  Zhuang  Manchu  Hui  Miao  Uighurs | 7  8  9  10  11  88 | Yi  Tujia  Mongolian  The Korean  Tibetan  Other nations |
| A4 | Your educational level | 1 Lack of formal schooling  2 Did not graduate from primary school  3 Graduated from primary school  4 Junior high school graduation  5 High school/technical school/technical school  6 Graduated from college  7 Undergraduate graduation  8 Postgraduate and above | | | |
| A5 | Your current marital status | 1  2  3  4 | Unmarried  Married  Cohabitation  Death of a spouse | 5 Divorce  6 Separation  88 Others | |
| A6 | Your occupation | 1 Agriculture, forestry, husbandry and fishing conservation  2 Production, transportation equipment operators  3 Business and service personnel  4 Persons in charge of enterprises and institutions  5 Officials and related personnel  6 Professional and technical personnel  7 A soldier  8 Other workers  9 Students  10 Unemployed  11 Housekeeping personnel  12 Retired persons | | | |
| A7 | What kind of health insurance do you currently have?  (Multiple options available) | 1 Basic medical insurance for urban workers  2 Free medical care  3 Medical insurance for urban residents  4 New rural cooperative medical care  5 Commercial medical insurance  6 Other  7 Didn't attend  99 Unclear | | | |

| **Part two Smoking status** | | | | | |
| --- | --- | --- | --- | --- | --- |
| Current smoking status | | | | | |
| B1 | Are you smoking **now**? | | 1 Yes, every day  2 Yes, but not every day ………… to  3 No …………………………… to | | B3  B14 |
| B2 | When did you start **smoking every day**? | | 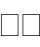 year | | |
| B3 How much of the following tobacco do you usually smoke?  Note to investigator: can't remember to fill in "-9", did not smoke to fill in "0" on the right. | | | | | |
| Type | | Amount of smoking (fill in 1 item only) | | | |
| a | Machine made cigarette | a1 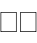 cigarettes/day | | a2 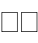 cigarettes/week | |
| b | Hand-rolled cigarette | b1 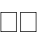 cigarettes/day | | b2 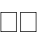 cigarettes/week | |
| c | Tobacco | c1 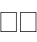 cigarettes/day | | c2 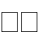 cigarettes/week | |
| d | Cigar | d1 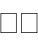 cigarettes/day | | d2 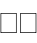 cigarettes/week | |
| e | Other - please specify  ____________ | e1 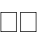 .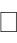 100g/day | | e2 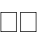 .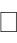 100g/week | |
| **Quit smoking behavior** | | | | | |
| B4 | Have you quit smoking in the past? | | 1 Yes  2 No ……………………………… .to | | B7 |
| B5 | Have you tried to quit smoking in the past 12 months? | | 1 Yes  2 No ………………………………to | | B7 |
| B6 | Have you used nicotine replacement therapy or other western medicine to try to quit smoking in the past 12 months? | | 1 Yes  2 No | | |
| B7 | Which of the following best fits your idea of quitting smoking? | | 1 Prepare to quit smoking in a month  2 Consider quitting smoking within 12 months  3 Quit smoking, but not within 12 months  4 Don't want to give up smoking  99 I don't know. | | |
| B8 | Have you seen any medical treatment in the past 12 months? | | 1 Yes  2 No …… ....... … ...... … ...... …… ... … to | | B10 |
| B9 | When you saw your doctor, did your health care provider advise you to quit smoking? | | 1 Yes  2 No | | |
| B10 | Have you seen a health warning on a cigarette packet in the past 30 days? | | 1 Yes  2 No …… ....... … ...... … ...... …… ... … to | | B12 |
| B11 | Have you seen health warnings on cigarette packets in the past 30 days made you think about quitting? | | 1 Yes  2 No  99 I don't know. | | |

| B12 | The last time you bought cigarettes for yourself, how many did you buy?  Note to investigators: Complete only one item. | a  b  c  d | 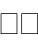 cigarettes  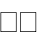 pack  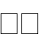carton  Never bought | ……… ... …… …to | B19 |
| --- | --- | --- | --- | --- | --- |
| B13 | How much did you spend on cigarettes that time?  Note to investigators: If you do not know, fill in "-9". | 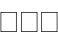 .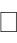 Yuan ... …… …to | | | B19 |
| B14 | Have you ever smoked **before**? | 1 Yes, every day  2 Yes, but not every day  3 No ……………………… ... to  99 I don't know. ……………………to | | | B19  B19 |
| B15 | How long have you stopped smoking?  (Investigator: Only those who quit smoking completely were included. Those who still smoked occasionally were not included. Note that only one item can be filled in) | a 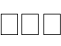 year  b 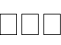 month  c 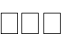 week  d 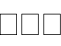 day | | | |
|  | Note to investigators: If B15 is less than 1 year (<12 month) **…......…......…...…** to  Otherwise **...…......…......……...………….......…......…......……...…** to | | | | B16  B19 |
| B16 | Have you seen any medical treatment in the past 12 months? | 1 Yes  2 No …… ....... … ...... … ...... …… ... … to | | | B18 |
| B17 | Did your doctor advise you to quit smoking? | 1 Yes  2 No | | | |
| B18 | Have you used nicotine alternative therapy or other western medicine to try to quit smoking in the past 12 months? | 1 Yes  2 No | | | |
| **Passive smoking** | | | | | |
| B19 | How many days per week are you usually exposed to secondhand smoke? | 1 No， 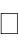 day  2 Barely …… ....... … ...... … ...... …… ... … to | | | B21 |
| B20 | If yes, how many days are there when the accumulated time exceeds 15 minutes? | 1 Yes， 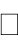 day 2 No | | | |
| B21 | Which of the following most accurately states your workplace's **indoor smoking** policy? | 1 Smoking is allowed anywhere  2 Smoking is allowed only in some indoor areas  3 Smoking is not allowed in all indoor areas  4 Work outside only  5 Work is at home/not at work  99 I don't know. | | | |

| B22.Have you been to any of the following places in the past 30 days? If so, is there anyone smoking indoors?  Note to investigators: If you have not been to the area in the past 30 days, skip the question "Does anyone smoke indoors?" | | | | |
| --- | --- | --- | --- | --- |
| Place | | 1. Have you been there?   1 Yes， 2 No | 1. Is there anyone smoking inside?   1 Yes， 2 No | |
| a  . | A government building or office  Note to investigators: This refers specifically to the offices of civil servants | 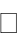 | 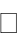 | |
| b  . | Medical institution | 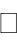 | 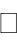 | |
| c  . | Primary and secondary schools  Note to investigators: the entire area, both inside and outside | 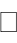 | 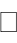 | |
| d  . | The restaurant | 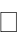 | 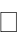 | |
| e  . | Any public transportation | 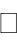 | 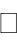 | |
| **Tobacco advertising** | | | | |
| B23. In the past 30 days, have you read any of the following media or places about the dangers of smoking or encouraging people to quit smoking?  Researchers note that participants should first be asked if they have been to any of these media or places in the past 30 days. If they have not, they should fill in the **"7"**. If they have, they should continue to ask if they have seen any information about the dangers of smoking or encouraging people to quit smoking. | | | | |
| A medium or place | | | | 1 Yes 2 No 7 NA |
| a  . | Newspaper or magazine | | | 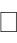 |
| b  . | TV | | | 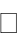 |
| c  . | Radio | | | 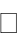 |
| d  . | Billboard | | | 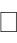 |
| e  . | A poster or promotional print | | | 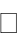 |
| f  . | Wall advertising in public places | | | 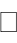 |
| g  . | The cinema | | | 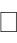 |
| h  . | The Internet | | | 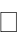 |
| i  . | A public transportation vehicle or station | | | 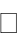 |
| j  . | Elsewhere, specify：  Note to the investigator: do not go to other places to fill in the "7", to see the relevant information to fill in the "1". | | | 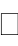 |

| B24：In the past 30 days, have you seen advertisements or signs promoting tobacco products in any of the following media or places?  Note to investigators: Respondents should first be asked if they have been exposed to these media or visited these places in the past 30 days. If they have not, they should directly enter "7". If they have, they should continue to ask if they have seen advertisements or signs promoting tobacco products. | | |
| --- | --- | --- |
| A medium or place | | 1 Yes 2 No 7 NA |
| a  . | Newspaper or magazine | 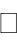 |
| b  . | TV | 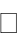 |
| c  . | Radio | 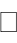 |
| d  . | Billboard | 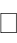 |
| e  . | A poster or promotional print | 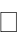 |
| f  . | Wall advertising in public places | 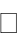 |
| g  . | The cinema | 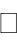 |
| h  . | The Internet | 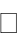 |
| i  . | A public transportation vehicle or station | 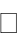 |
| j  . | Newspaper or magazine | 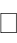 |
| k  . | Elsewhere, specify：  Note to the investigator: do not go to other places to fill in the "7", to see the relevant information to fill in the "1". | 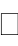 |

| Part Three Alcohol consumption | | | |
| --- | --- | --- | --- |
| C1 | Have you had any alcohol in the last 12 months? | 1 Yes, in the last 30 days  2 Yes, about 30 days ago  3 No …………………… … to | D1 |
| C2 | How often have you consumed alcohol in the past 12 months?  (Read-out option required) | 1 Every day  2 5-6 day/week  3 3-4 day/week  4 1-2 day/week  5 1-3 day/month  6 Less than 1 day/month | |

| Please answer: In the past 12 months, how often do you usually drink the following wines? How much do you usually drink each day?  Note to the investigator: if you can't remember clearly, fill in "- 9" on the right before the decimal point. If you don't drink, don't fill in the frequency and amount of drinking | | | | | | |
| --- | --- | --- | --- | --- | --- | --- |
| C3 |  | 1. Drinking or not   1 Yes， 2 No | b Drinking frequency (fill in only one item) | | | The amount of alcohol consumed in a typical day in the past 12 months |
|  |  |  | b1  day/week | b2  day/month | b3  day/year |  |
|  | a. Baijiu (≥42 degrees) | 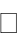 | 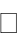 | 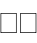 | 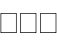 | 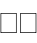 .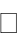 g |
|  | b. Baijiu (<42 degrees) | 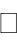 | 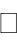 | 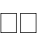 | 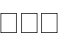 | 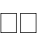 .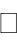 g |
|  | c. Beer (520ml/bottle) | 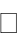 | 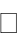 | 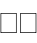 | 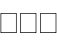 | 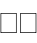 .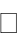 g |
|  | d. Yellow wine | 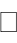 | 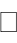 | 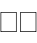 | 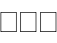 | 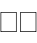 .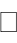 g |
|  | e. Rice wine | 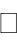 | 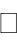 | 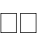 |  | . g |
|  | f. Wine |  |  |  |  | . g |
|  | g. Barley wine |  |  |  |  | . g |
| C4 | 1. For men:   In the past 12 months, how often did you drink more than 2.5 liang of high alcohol Baijiu, or 3.5 liang of low alcohol Baijiu, or 3 bottles of beer, or 5 cans of beer, or 7.5 liang of yellow rice wine, or 1 kg and a half of wine, or 3 kg of highland barley wine at a time? | | | 1 Every day or almost every day (≥ 5 days/week)  2 1-4 days/week  3 1-3 days/month  4 Less than 1 day/month  5 Never | | |
|  | 1. For women:   In the past 12 months, how often did you drink more than 2 liang of high alcohol Baijiu, or 3 liang of low alcohol Baijiu, or 2.5 bottles of beer, or 4 cans of beer, or 6 liang of yellow rice wine, or 1 kg of 2 liang of wine, or 2.5 kg of highland barley wine at a time? | | | 1 Every day or almost every day (≥ 5 days/week)  2 1-4 days/week  3 1-3 days/month  4 Less than 1 day/month  5 Never | | |
| C5 | How often have you been drunk in the past 12 months?  Note to the investigator: the answers must be read out for the respondents to choose | | | 1 Every day or almost every day (≥ 5 days/week)  2 1-4 days/week  3 1-3 days/month  4 Less than 1 day/month  5 Never | | |

| Part Four Diet | | | | |
| --- | --- | --- | --- | --- |
| D1 | How many meals do you usually eat a day in the past 12 months? | meals | | |
|  | | Dining place | | |
|  |  | a Home | b Canteen | c Restaurant |
| D2 | In the past 12 months, how many days do you usually have breakfast at different eating places in a week? | day | day | day |

|  | | | | a Home | | Dining place  b Canteen | | c Restaurant | |
| --- | --- | --- | --- | --- | --- | --- | --- | --- | --- |
| D3 | In the past year, how many days do you usually have lunch at different eating places in a week? | | | day | | day | | day | |
| D4 | In the past year, how many days do you usually have dinner at different eating places in a week? | | | day | | day | | day | |
| Please recall whether you have eaten the following foods in the past 12 months, and estimate the frequency and quantity of various foods. | | | | | | | | | |
|  | | a Whether to eat  1 Yes，2 No | b Frequency of consumption | | | | | | Average consumption per time |
|  |  |  | b1  times/day | | b2  times/week | b3  times/month | b4  times/year | |  |
| D5 | Cereal foods (recorded by raw weight) |  |  | |  |  |  | | .g |
| D6 | tubers |  |  | |  |  |  | | .g |
| D7 | pork |  |  | |  |  |  | | .g |
| D8 | Meat of cattle, sheep, etc. |  |  | |  |  |  | | .g |
| D9 | Chicken duck goose and other poultry meat |  |  | |  |  |  | | .g |
| D10 | Aquatic products |  |  | |  |  |  | | .g |
| D11 | Fresh vegetables |  |  | |  |  |  | | .g |
| D12 | Fresh fruit |  |  | |  |  |  | | .g |
| D13 | Freshly squeezed vegetable juice (250ml/ cup) |  |  | |  |  |  | | .cup |
| D14 | eggs |  |  | |  |  |  | | . |
| D15 | Dairy products |  |  | |  |  |  | | .g |
| D16 | Soy products |  |  | |  |  |  | | .g |
| D17 | Fried food |  |  | |  |  |  | | .g |
| D18 | Fruit and vegetable juice/fruit-flavored drink (250ml/ cup) |  |  | |  |  |  | | .cup |

|  | | | 1. Whether to eat   1 Yes，2 No | b Frequency of consumption | | | | Average consumption per time | |
| --- | --- | --- | --- | --- | --- | --- | --- | --- | --- |
|  |  |  |  | b1  times/day | b2  times/week | b3  times/month | b4  times/year |  |  |
| D19 | | Carbonated beverage (250ml/cup) |  |  |  |  |  | .cup | |
| D20 | | Cakes |  |  |  |  |  | .g | |
| D21 | | Pickles |  |  |  |  |  | Don't "fill in" | |
| D22 | | pickled cabbage |  |  |  |  |  |  |  |
| D23 | | Fermented bean curd |  |  |  |  |  |  |  |
| D24 | | Tea |  |  |  |  |  |  |  |
| D25 | | Coffee |  |  |  |  |  |  |  |
| D26 | | Animal innards |  |  |  |  |  |  |  |
| D27 | | Nutrient supplement |  |  |  |  |  |  |  |
| D28 | | Do you think eating more salt will affect your health? | | | 1 Yes  2 No ... … ... … ....... ……… to  99 Unclear ... … ....... ……… to | | | | D30  D30 |
| D29 | | Which of the following diseases do you think will be aggravated or caused by eating too much salt? (**Multiple options available**) | | | 1 Hypertension 5 Uncorrelated  2 Diabetes 88 Other  3 Cataract 99 Unclear  4 Arthritis | | | | |
| D30 | | Would you be willing to eat less salt if you knew it was bad for you? | | | 1 Yes  2 No  3 Indifferent  99 Unclear | | | | |
| D31 | Are you currently cutting down on salt? | | | | 1 Yes  2 No … ... … ........ … ... … ...... …to | | | | D33 |
| D32 | If yes, what specific measures have you taken?  (**Multiple options available**) | | | | 1 Reduce eating out  2 Use less salt when cooking food  3 Eat less food with high salt content  4 No extra salt when eating at the table  5 Use salt limiting tools, such as salt control spoon | | | | |
| D33 | What foods do you eat salt from?  (**Multiple choices are allowed**) | | | | 1 Cooking salt, such as edible salt, soy sauce, etc.  2 Preserved foods, such as pickles and sauerkraut  3 Processed foods, such as sausage, canned food, etc.  4 Fast food, such as instant noodles  5 Snacks, such as French fries  88 Other | | | | |

| **Part Five Physical activity** | | | |
| --- | --- | --- | --- |
| The following questions are about various physical activities. Please answer the questions: | | | |
| Physical activity for work, agriculture and housework | | | |
| E1 | Are there any high-intensity activities in your work, farm work and household activities that last more than 10 minutes?  Note to investigators: Show the physical activity classification table. | 1 Yes  2 No ……………… ... …to | E4 |
| E2 | In your work, farm work and household activities, how many days in a week do you usually have the above high intensity activities? | day | |
| E3 | In your work, farm work and household activities, how much time do you usually spend on the above-mentioned high-intensity activities in a day?  Note to the investigator: if the time of each activity is less than 10 minutes, it will not be counted. | hour minutes | |
| E4 | Are there any moderate intensity activities in your work, farm work and household activities that last more than 1 minute?  Note to investigators: Show the physical activity classification table. | 1 Yes  2 No ………………to | E7 |
| E5 | In your work, farm work and household activities, how many days in a week do you usually do the above moderate intensity activities? | day | |
| E6 | In your work, farm work and household activities, how much time do you usually spend on the above moderate intensity activities in a day?  Note to the investigator: if the time of each activity is less than 10 minutes, it will not be counted. | hour minutes | |
| Transportation physical activity  The following questions do not include the above-mentioned agricultural physical activities and work and household physical activities. | | | |
| E7 | Do you walk or ride a bicycle for at least 10 minutes when you go out? | 1 Yes  2 No ……………… ... …to | E10 |
| E8 | U How many days a week do you usually walk or bike for at least 10 minutes when you're out and about? | day | |
| E9 | How long do you usually walk or ride a bike in a day? | hour minutes | |

| Leisure physical activity  The following issues do not include agricultural, work, household and transportation physical activities mentioned above. | | | |
| --- | --- | --- | --- |
| E10 | Do you perform high-intensity activities that last at least 10 minutes and cause significant increases in respiration and heartbeat? Such as long-distance running, swimming, playing football, etc.  Note to investigators: Show the physical activity classification table. | 1 Yes  2 No …………… to | E13 |
| E11 | U How many days in a week do you usually have these high-intensity sports or leisure activities? | day | |
| E12 | U How much time do you spend on the above high intensity sports or leisure activities in a day? | hour minutes | |
| E13 | Do you have moderate intensity sports and leisure activities that last at least 10 minutes and cause a slight increase in respiration and heartbeat? For example, walking fast and playing Taijiquan.  Note to investigators: Show the physical activity classification table. | 1 Yes  2 No …………… to | E16 |
| E14 | How many days in a week do you usually do the above moderate intensity sports or leisure activities? | day | |
| E15 | How long do you usually spend on the above moderate intensity sports or leisure activities in a day?  Note to the investigator: if the time of each activity is less than 10 minutes, it will not be counted. | hour minutes | |

| Total static behavior | | |
| --- | --- | --- |
| E16 | How much time do you spend sitting, leaning or lying down in a day? (Including time for sitting, working, studying, reading, watching TV, using the computer, resting and other static behaviors, but excluding sleeping time) | hour minutes |
| Static behavior in spare time | | |
| E17a | How much time do you spend watching TV in your spare time? | hour minutes |
| E17b | In your spare time, how much time do you use the computer on average every day? | hour minutes |
| E17c | How much time do you spend on reading (paper books) in your spare time? | hour minutes |
| E17d | How much time do you spend on playing video games (excluding computer games) in your spare time? | hour minutes |

| Internet behavior | | | |
| --- | --- | --- | --- |
| E18a | How much time do you spend on surfing the Internet every day (using computers, netbooks, mobile phones and other tools to surf the Internet)? | 1 hour minutes  2 Never ………………… to | E19 |
| E18b | What do you spend most time online for? | 1 Job needs  2 Access to information (not required for work)  3 Chat (instant messaging tool)  4 Online community and forum discussion  5 Network game  6 Watch video programs and listen to music  7 Shopping  88 Other | |
| Sleep behavior | | | |
| E19 | How much time do you sleep in a day? | hour minutes | |

| Part Six Weight, blood pressure, blood sugar, blood lipid and other information | | | | |
| --- | --- | --- | --- | --- |
| **F1** Weight and its control | | | | |
| F1a | When did you measure your weight last? | | 1 Never  2 Within 7 days  3 Within 1month  4 Within 3 months  5 Within 6 months  6 Within 12 months  7 12 months ago  99 Unclear | |
| F1b | Do you know what body mass index (BMI) is? | | 1 Yes  2 No | |
| F1c | Have you taken any measures to control your weight in the past 12 months? | | 1 Take measures to lose weight  2 Take measures to keep weight  3 Take measures to lose weight ……. …to  4 No action was taken………………. …to | F2a  F2a |
| F1d | What are your ways to control or lose weight? (Multiple choices are allowed) | | 1 Diet control  2 Physical exercise  3 Medicine  88 Other | |
| **F2** Blood pressure and its control | | | | |
| F2a | Have your parents ever suffered from high blood pressure? | 1 Yes  2 No  99 Unclear | | |

| F2b | When did you measure your blood pressure last? | 1 Within 7 days  2 Within 1month  3 Within 6 months  4 Within 12 months  5 12 months ago  6 Never ………………………… .to  99 Unclear | F3a |
| --- | --- | --- | --- |
| F2c | Have you ever been diagnosed with hypertension by a doctor? | 1 Yes  2 No …………………………to | F2h |
| F2d | Have you taken any measures to control your blood pressure? | 1 Yes  2 No …………………………to | F2g |
| F2e | What measures have you taken to control your blood pressure? (Multiple choices are allowed) | 1 Take medicine according to doctor's instructions  2 Take medicine when there are symptoms  3 Diet control  4 Taking Exercise  5 Blood pressure monitoring  88 Other | |
| F2f | Have you taken any antihypertensive drugs in the last 2 weeks? | 1 Yes  2 No | |
| F2g | In the past year, doctors in primary health service institutions have provided you with the number of blood pressure measurements (excluding examinations when sick) | times  Note to the investigator: fill in "- 9" if respondent can't remember clearly | |
| F2h | In the past year, did the first doctor you received measure your blood pressure when you went to the hospital? | 1 Yes  2 No  3 Sometimes  4 Never  99 Unclear | |
| **F3** **Blood glucose and its control** | | | |
| F3a | Have your parents ever suffered from diabetes? | 1 Yes  2 No  99 Unclear | |
| F3b | How long has it been since your last blood glucose measurement? | 1 Within 6 months  2 Within 12 months  3 12 months ago  4 Never ………………………to  99 Unclear | F4a |
| F3c | Have you ever been diagnosed with diabetes by your doctor? | 1 Yes  2 No ………………………to | F4a |
| F3d | Have you taken any measures to control blood sugar? | 1 Yes  2 No ………………………to | F3f |
| F3e | What measures have you taken to control blood sugar?  (Multiple choices are allowed) | 1 Oral medicine  2 Insulin injection  3 Diet control  4 Taking Exercise  5 Blood glucose monitoring  88 Other | |

| F3f | How many blood glucose follow-up visits have you received from primary health service institutions in the past year? | times  Note to the investigator: fill in "- 9" if respondent can't remember clearly | | | |
| --- | --- | --- | --- | --- | --- |
| **F4** Blood lipid and its control | | | | | |
| F4a | Have you been diagnosed as dyslipidemia by your doctor? | 1 Yes  2 No ………………… ………to | | | F5a |
| F4b | Have you taken any measures to control blood lipids? | 1 Yes  2 No …………………………to | | | F5a |
| F4c | What measures have you taken to control blood lipids?  (Multiple choices are allowed) | 1 Take medicine according to doctor's instructions  2 Diet control  3 Taking Exercise  5 Blood lipid monitoring  88 Other | | | |
| **F5** Cardio cerebral vascular events | | | | | |
| F5a | Have you had any myocardial infarction in the past 12 months? | | 1 Yes  2 No | | |
| F5b | Have you had any stroke in the past 12 months? | | 1 Yes  2 No | | |
| **F6** Other chronic diseases | | | | | |
| F6a | Do you have chronic obstructive pulmonary disease (such as chronic bronchitis, emphysema)? | | 1 Yes  2 No | | |
| F6b | Do you have asthma? | | 1 Yes  2 No | | |
| F6c | Malignant tumors (including malignant tumors of the whole body and benign tumors of the brain)? | | 1 Yes  2 No | | |
| **F7** **Respiratory system condition (only for respondents aged 40 and above)**  **If<40 years old .............................................................................................................to** | | | | | **G1** |
| F7a1 | In the past 12 months, have you often coughed when you woke up in the morning? | | | 1 Yes  2 No | |
| F7a2 | In the past 12 months, have you often coughed during the day or at night? | | | 1 Yes  2 No | |
| If either of the above two questions F7a1 and F7a2 is answered "Yes", continue; if both are answered "No", skip to F7b1 | | | | | |
| F7a3 | Do you cough like this for three months or more every year? | | | 1 Yes  2 No | |
| F7b1 | In the past 12 months, did you cough up sputum in the morning? | | | 1 Yes  2 No | |
| F7b2 | In the past 12 months, do you often cough up sputum during the day or at night? | | | 1 Yes  2 No | |
| If any of the above questions F7b1 and F7b2 is answered as "Yes", continue; if all the answers are answered as "No", skip to F7c | | | | | |
| F7b3 | Do you expectorate for three months or more every year? | | | 1 Yes  2 No | |
| F7c | Have you ever had a pulmonary function test? | | | 1 Yes  2 No  99 Unclear | |

| Part VII Health status | | | |
| --- | --- | --- | --- |
| **G1** General health | | | |
| G1a | What do you think of your health? | 1 Very good  2 Good  3  Commonly  4 Bad  5 Very bad | |
| G1b | In the past 30 days, how many days have you been in poor health due to illness? | day  -9 can't remember clearly | |
| G1c | In the past 30 days, how many days have you been in poor health due to injury? | day  -9 can't remember clearly | |
| G1d | In the past 30 days, how many days have you been in poor health due to stress, depression or emotional problems? | day  -9 can't remember clearly | |
| **G2** 健康体检 | | | |
| G2a | How long has it been since your last physical? (Excluding medical examination during medical treatment)  Note to the investigator: only one item shall be filled in for month and year. | 1 month  2 year  3 Never had a physical examination....to | G3 |
| G2b | What is the reason for your physical examination? | 1 Free of charge  2 Free in the community  3 Self care  88 Other | |
| **G3** Screening for cervical cancer and breast cancer in women (women only)  If male ......................................................... ...... ...... .............................................................to | | | G4a |
| G3a | Have you ever had a cervical smear? | 1 Yes  2 No ………………… ... …to  99 Unclear …… ………………to | G3c  G3c |
| G3b | If yes, when was the last inspection?  Note to the investigator: fill in "00" in less than one year. | years ago. | |
| G3c | Have you ever had a breast examination? | 1 Yes  2 No ……………………to  99 Unclear …………………. …to | G4a  G4a |
| G3d | If yes, when was the last inspection?  Note to the investigator: fill in "00" in less than one year. | years ago. |  |
| **G4** Mental health | | | |
| Please answer the following questions as truthfully as possible about your own feelings, emotions or experiences. All information will be kept confidential. | | | |
| G4a | In the last two weeks, do you think you are not interested in doing anything? | 1 Never  2 A few days, but not many  3 More than one week  4 Almost every day  99 Unclear  97 Refuse to answer | |

| G4b | In the last 2 weeks, do you feel depressed, depressed, or hopeless? | 1 Never  2 A few days, but not many  3 More than one week  4 Almost every day  99 Unclear  97 Refuse to answer | |
| --- | --- | --- | --- |
| G4c | In the last 2 weeks, have you been unable to sleep, unable to sleep soundly, or sleeping too much? | 1 Never  2 A few days, but not many  3 More than one week  4 Almost every day  99 Unclear  97 Refuse to answer | |
| G4d | Do you feel tired and listless in the last 2 weeks? | 1 Never  2 A few days, but not many  3 More than one week  4 Almost every day  99 Unclear  97 Refuse to answer | |
| G4e | Have you had no appetite or eaten too much in the last 2 weeks? | 1 Never  2 A few days, but not many  3 More than one week  4 Almost every day  99 Unclear  97 Refuse to answer | |
| G4f | In the last 2 weeks, have you been dissatisfied with yourself, or feel that you are a loser, or let you or your family down? | 1 Never  2 A few days, but not many  3 More than one week  4 Almost every day  99 Unclear  97 Refuse to answer | |
| G4g | In the last 2 weeks, do you feel that you can't concentrate on things, such as reading books, newspapers or watching TV? | 1 Never  2 A few days, but not many  3 More than one week  4 Almost every day  99 Unclear  97 Refuse to answer | |
| G4h | In the last 2 weeks, do you feel that you have become slow in action or speaking, causing others to pay attention to you, or on the contrary, you are restless and fidgety, and you are more likely to walk around than usual? | 1 Never  2 A few days, but not many  3 More than one week  4 Almost every day  99 Unclear  97 Refuse to answer | |
| G4i | In the last 2 weeks, have you ever thought of committing suicide or hurting yourself? | 1 Never  2 A few days, but not many  3 More than one week  4 Almost every day  99 Unclear  97 Refuse to answer | |
| If G4a-G4i is filled with "none", then……………… .......................................................................to | | | G4k |
| G4j | If you have the above questions (G4a-G4i), do they affect your daily work, family life and interpersonal relationship? | 1 No impact  2 Some impacts  3 Very influential  4 Great impact  99 Unclear  97 Refuse to answer | |

| G4k | What is your relationship with your family (such as parents, spouse, children) in the past 12 months? | 1 Very good  2 Good  3Commonly  4 Bad  5 Very bad  6 Living alone  97 Refuse to answer |
| --- | --- | --- |
| G4l | In the past 12 months, have you encountered anything that has hit you hard? | 1 Yes  2 No  97 Refuse to answer |
| G4m | In the past 12 months, in general, are you satisfied with your life? | 1 Very satisfied  2 Satisfied  3 Commonly  4 Dissatisfied  5 Very dissatisfied  97 Refuse to answer |

| **Part VIII Information related to transient cerebral ischemia** | | | |
| --- | --- | --- | --- |
| H1 | Have you heard of "transient ischemic attack/TIA/transient ischemic attack"? | 1 Heard of  2 Never heard of it...... … ......... ... ...… to | **H6** |
| H2 | The abbreviation of "transient ischemic attack" is "TIA", also known as "transient ischemic attack". Do you know the relationship between transient ischemic attack and stroke? | 1 TIA is a stroke  2 TIA is a symptom of stroke  3 TIA has nothing to do with stroke  88 Other  99 Don't know ........... ........ ..... ..... ..... ..... ....to | **H6** |
| H3 | Where do you get the information of "transient ischemic attack/TIA"? (**Multiple choices are allowed**) | 1 Obtained when seeing a doctor due to transient ischemic attack or stroke  2 Obtained when seeing a doctor for other diseases  3 Not from medical treatment, but from relevant professionals in the medical industry  4 Obtained from TV, radio, newspapers, internet and other media  5 Obtained from family, friends, etc.  6 Respondents: medical and health professionals  88 Other： _____________________  99 Can't remember clearly | |
| H4 | What are the symptoms of "transient ischemic attack/TIA"?  (**Multiple choices are allowed**) | 1 Sudden numbness and weakness of one side of upper and lower limbs, numbness of one side of face, and inclination of corners of mouth.  2 Suddenly, the eyes of one or both eyes become black, and the vision is unclear or unable to see; The vision of the same side of both eyes darkens and disappears.  3 Sudden speech disorder or hearing disorder.  4 A sudden imbalance.  88 Other： _____________  99 Unclear | |

| H5 | Have you ever been diagnosed with transient ischemic attack/TIA by your doctor? | | | | 1 Yes ……. ...… to  2 No  99 Unclear | | | | | **H9** |
| --- | --- | --- | --- | --- | --- | --- | --- | --- | --- | --- |
| H6 | Have you ever had the following symptoms and how long did they last?  **Note to the investigator:** List the following items to the interviewee and record the patient's answers in the following table. | | | | | | | | | |
|  | Symptom | Are there any symptoms?  1 Yes  2 No  -9 can't remember clearly | Symptom duration  (Fill in only one item, and fill in "1" in the appropriate box) | | | | | | | |
|  |  |  | ≤ 10 minutes | | | 10 minutes to 1 hour | ≥ 1 hours | | Unclear | |
|  | a. Sudden numbness and weakness of one side of upper and lower limbs, numbness of one side of face, and inclination of corners of mouth. |  |  | | |  |  | |  | |
|  | b. Sudden blackness of one or both eyes, unclear vision or inability; The vision of the same side of both eyes darkens and disappears. |  |  | | |  |  | |  | |
|  | c. A sudden impediment to speaking or listening |  |  | | |  |  | |  | |
|  | d. Sudden balance disorder. |  |  | | |  |  | |  | |
|  | If "2 No" or "- 9 Unclear" is selected for all the above symptoms………to | | | | | | | Questionnaire Part IX | | |
| H7 | Have you seen a doctor when the above symptoms appear? | | | 1 Yes  2 No ………………to  99 Can't remember clearly. …… . …… . ………to | | | | Questionnaire part IX  Questionnaire part IX | | |
| H8 | What was your doctor's diagnosis at that time  Note to the investigator: List the following items to the interviewee | | | 1 TIA  2 cervical spondylopathy  3 Migraine  4 No diagnosis  88 Other： _________  99 Unclear | | | | To questionnaire part IX | | |
| H9 | Which of the following symptoms do you have during the "transient ischemic attack/TIA" attack? | | | | | | | | | |
|  | Symptom | Are there any symptoms?  1 Yes  2 No  -9 can't remember clearly | Symptom duration  (Fill in only one item, and fill in "1" in the appropriate box) | | | | | | | |
|  |  |  | ≤ 10 minutes | | | 10 minutes to 1 hour | ≥ 1 hours | | Unclear | |
|  | a. Sudden numbness and weakness of one side of upper and lower limbs, numbness of one side of face, and inclination of corners of mouth. |  |  | | |  |  | |  | |

|  | Symptom | Are there any symptoms?  1 Yes  2 No  -9 can't remember clearly | Symptom duration  (Fill in only one item, and fill in "1" in the appropriate box) | | | |
| --- | --- | --- | --- | --- | --- | --- |
|  |  |  | ≤ 10 minutes | 10 minutes to 1 hour | ≥ 1 hours | Unclear |
|  | b. Sudden blackness of one or both eyes, unclear vision or inability; The vision of the same side of both eyes darkens and disappears. |  |  |  |  |  |
|  | c. Sudden speech disorder or hearing disorder. |  |  |  |  |  |
|  | d. Sudden imbalance. |  |  |  |  |  |
|  | e. Other symptoms 1 (specific symptoms, do not read, please record) _____________ |  |  |  |  |  |
|  | f. Other symptoms 2 (specific symptoms, do not read, please record). |  |  |  |  |  |
|  | g. Other symptoms 3 (specific symptoms, do not read, please record). |  |  |  |  |  |
| H10 | Please recall your last "transient ischemic attack/TIA" attack. How long did you go to see a doctor after the symptoms appeared? | | | 1 Within 24 hours  2 25-48 hours  3 2 to 7 days  4 Exceed 7 days  5 No medical treatment ………………to  99 Unclear | | **H12** |
| H11 | Please recall your last "transient ischemic attack/TIA" attack. Do you have the following items in the prescription or order given by your doctor? (**Multiple choices are allowed**)  Note to the investigator: List the following items to the interviewee. | 1 Aspirin  2 PLAVIX (Clopidogrel/Tai chi)  3 Dipyridamole  4 Warfarin  5 Traditional Chinese medicine, Chinese patent medicine  6 No drug prescription  7 Carotid Surgery/Carotid Stents  88 Other processing (please record). ________________  99 Unclear | | | | |

| H12 | What kind of doctor is the first doctor after "transient ischemic attack/TIA"?  Note to the investigator: List the following items to the interviewee and record the patient's answers. | 1 Rural community doctors  2 Urban community doctors  3 Doctor of traditional Chinese medicine  4 Private Clinic doctor  5 Physician of neurology………………to  6 Emergency doctor…………………to  7 General outpatient ………………to  99 Unclear ………………………………to | H14  H14  H14  Questionnaire part IX |
| --- | --- | --- | --- |
| H13 | Did the community doctor of the first community health service station let you go to a secondary or higher hospital to see a specialist for further treatment? | 1 Yes  2 No  99 I don’t know. | |
| H14 | If your first doctor suggested you go to a specialist for further consultation, what kind of specialist would you be? | 1 Physician of neurology  2 Physician of the heart  3 Ophthalmologist  4 Other doctors (please record)  99 Unclear | |

| Part IX Oral health | | | |
| --- | --- | --- | --- |
| I1 | How long did you last see the dental distance? | 1 Less than 1 year  2 1-2 year  3 3-4 year  4 5 years and above  5 Never  99 Can't remember clearly | |
| I2 | How long has it been since you last washed your teeth? | 1 Less than 1 year  2 1-2 year  3 3-4 year  4 5 years and above  5 Never  99 Can't remember clearly | |
| I3 | How many of your real teeth were pulled out because of tooth decay or periodontal disease?  **Note to the investigato**r: "00" is not filled in | teeth | |
| I4 | This question is answered by residents over 50 years old.  How many real teeth do you have?  Note to investigators: "- 9" is not clear | teeth | |
| I5 | How often do you brush your teeth? | 1 More than 2 times/day  2 2 times/day  3 1 times/day  4 Less than 1 times/day  5 Never …………………to | I7 |

| I6 | Do you use fluoride toothpaste now? | 1  2  3 | Used  Not used  Unclear | |
| --- | --- | --- | --- | --- |
| I7 | Have you had bleeding gums in the past 12 months? | 1  2  3 | Often  Sometimes  No …………………………to | J1 |
| I8 | What do you usually do when your gums bleed?  (Multiple choices are allowed) | 1  2  3  4  5  6  7 | Pay attention to brushing your teeth  Gargle with clean water  Rinse with salt water  Taking medicine  Ask a dentist for treatment  Other methods  Never mind, I don't care | |

| **Part X Injuries and risk factors** | | | |
| --- | --- | --- | --- |
| Road traffic injuries | | | |
| J1 | In the past 30 days, have you worn a helmet when driving or riding a motorcycle? | 1 Often  2 Sometimes  3 Seldom  4 No  5 Didn't drive/ride a motorcycle in the past 30 days | |
| J2 | Have you ever taken a motor vehicle in the past 30 days? | 1 Yes  2 No……………………to | J4 |
| J3 | In the past 30 days, when you were in the front row of a motor vehicle, did someone remind you to wear a seat belt? | 1 Yes  2 No  3 Didn't sit in the front seats  4 The motor vehicle you are riding does not have a seat belt | |
| J4 | Have you ever driven a motor vehicle in the past 30 days? | 1 Yes  2 No………………………… .......to | J9 |
| J5 | In the past 30 days, have you worn a seat belt while driving? | 1 Often  2 Sometimes  3 Seldom  4 No | |
| J6 | Have you had at least one experience of drunk driving in the past 30 days? | 1 Yes  2 No  97 Refuse to answer | |
| J7 | In the past 30 days, have you ever driven continuously for more than 4 hours without rest? | 1 Yes  2 No  97 Refuse to answer | |
| J8 | Do you have a motor vehicle driver's license? | 1 Yes  2 No  97 Refuse to answer | |

| The following questions relate to injuries you have experienced in the past 12 months.  (Any one of the following three conditions is considered as an injury event: (1) Go to the medical unit for diagnosis and treatment, and diagnose a certain type of injury; (2) Emergency treatment by others due to injury; (3) Rest for more than half a day due to injury) | | | | | | | | | |
| --- | --- | --- | --- | --- | --- | --- | --- | --- | --- |
| J9 | In the past 12 months, have you ever experienced injury events, such as traffic accidents, falls, animal bites, knife wounds, scalds, etc.  **Note to investigators**: List the causes of injury in question J10. | | 1 Yes  2 No …………………… …............to  99 Unclear………… ……… ... … .to  97 Refuse to answer…………… ... ……to | | | | | | End  End  End |
| **Note to investigators:** each injury must be recorded. | | Occurrence of each injury | | | | | | | |
|  |  | ① | | ② | ③ | ④ | ⑤ | ⑥ | |
| J10 | The cause of the injury?  1 Road traffic injuries  2 Dropping  3 Blunt force injury  4 Firearm wound  5 Knife/sharp instrument injury  6 Burn and scald  7 Asphyxiation  8 Drowning  9 Poisoning  10 Animal injury  11 Sexual Assault.  88 Other  99 Unclear | 1  2  3  4  5  6  7  8  9  10  11  88  99 | | 1  2  3  4  5  6  7  8  9  10  11  88  99 | 1  2  3  4  5  6  7  8  9  10  11  88  99 | 1  2  3  4  5  6  7  8  9  10  11  88  99 | 1  2  3  4  5  6  7  8  9  10  11  88  99 | 1  2  3  4  5  6  7  8  9  10  11  88  99 | |
| J11 | Did you seek medical attention?  Yes  1 Outpatient or emergency  2 Hospitalization  No  3 Slight, no need to see a doctor  4 Need treatment, unable to achieve  (For example, due to the remote location of medical treatment and poor family) | 1  2  3  4 | | 1  2  3  4 | 1  2  3  4 | 1  2  3  4 | 1  2  3  4 | 1  2  3  4 | |

Survey end time (24-hour system)： hours minutes

Local projects transferred by the central government

**Chronic Disease Surveillance in China (2010)**

**Body measurement record form**

| Personal code： |
| --- |

| Height, weight and waist circumference inquiry | | | |
| --- | --- | --- | --- |
| Hello, we will ask you some questions about your height and weight. | | | |
| K1 | Do you know your height? | 1 Yes  2 No........................................to | K3 |
| K2 | If yes, what is your height? | . cm | |
| K3 | Do you know your weight? | 1 Yes  2 No........................................to | K5 |
| K4 | If yes, what is your weight? | . kg | |
| K5 | Do you know your waist circumference? | 1 Yes  2 No........................................to | M1 |
| K6 | If yes, what is your waist circumference? | . cm |  |
| Body measurement | | | |
| Hello, now we will measure your height, weight, waist circumference and blood pressure. Please cooperate. | | | |
| M1a | Surveyor Name 1 | ________________________ | |
| M1b | Surveyor Name 2 | ________________________ | |
| M2 | **Height**  Note to the investigator: If the height exceeds the range, record - 9. | . (cm)  . (kg) | |
| M3 | **Weight**  Note to the investigator: if the weight exceeds the range, record - 9. |  |  |
| Waist | | | |
| M4 | Waist | . (cm) | |

CNCDS2010-PM

| Blood pressure and heart rate | | | |
| --- | --- | --- | --- |
| M5 | Surveyor name | ______________________________ | |
| M6 | Code of sphygmomanometer |  | |
| M7a | First reading.  Note to the investigator: measure and record the blood pressure for the first time after 15 minutes of rest, and measure the blood pressure and heart rate for the second time after 1 minute of rest | Systolic pressure | (mmHg) |
| M7b |  | Diastolic pressure | (mmHg) |
| M7c |  | Heart rate | times/minute |
| M8a | 2nd reading  Note to the investigator: record the second measurement result, and measure the blood pressure and heart rate for the third time after the measured object has a rest of 1 minute | Systolic pressure | (mmHg) |
| M8b |  | Diastolic pressure | (mmHg) |
| M8c |  | Heart rate | times/minute |
| M9a | 3rd reading  Record the third measurement result | Systolic pressure | (mmHg) |
| M9b |  | Diastolic pressure | (mmHg) |
| M9c |  | Heart rate | times/minute |
