# Supplementary material for: Relationship between socioeconomic status and hypertension incidence among adults in southwest China: a population-based cohort study
Source: BMC Public Health. 2024 May 2;24:1211. doi: 10.1186/s12889-024-18686-5 (PMC11064324; doi:10.1186/s12889-024-18686-5)
Supplement: Supplementary file 1 — Supplementary Material 1. [file 12889_2024_18686_MOESM1_ESM.zip › Follow up questionnaire.docx]

1、 Family Questionnaire

Part II Questionnaire description

| Import Personal Code |
| --- |

According to Article 15 of Chapter III of the Statistics Law of the People's Republic of China, "Individual survey data belonging to private individuals and families shall not be disclosed without their consent".

Chronic disease and nutrition surveillance in Guizhou province.

Family Questionnaire

| Name of respondents: **import** respondents Phone 1: **import** (can be changed or supplemented) | |
| --- | --- |
| Respondent telephone 2 (landline): **import** (can be changed or supplemented) Respondent telephone (mobile phone) 3: **impor**t (can be changed or supplemented) | |
| Contact person of survey object: **import** (can be changed or supplemented) | |
| Contact Phone 1: **Import** (can be changed or added) Contact Phone 2: **Import** (can be changed or added) | |
| Survey point name (county/district): **import** | |
| Township/street name: **import** | |
| Village/neighborhood committee name: **import** | |
| Home address: **import** (can be changed or supplemented) | |
| Investigator Name: | Date: automatically generated |

Note: if the phone number is unknown, fill in - 8

| Contact record | | | | |
| --- | --- | --- | --- | --- |
|  | | 1. First time | b. Second time | c. Third time |
| HC1 | Date | __month__ day | __month__ day | __month__ day |
|  | Fill in the details of each contact with the respondents. The contact information can be phone number, home appointment, etc.  Note that if the date of contact appointment is different from the date of family questionnaire, please fill in the date of contact appointment. If the survey household is contacted on October 20 and notified that the survey team will enter the household on October 22, the contact date should be "October 20" | | | |
| HC2 | Contact time  1 Weekday daytime  2 Weekday evening  3 Holiday | 1  2  3 | 1  2  3 | 1  2  3 |
|  | For the respondents who failed to contact for three times, the time for three times of contact should be different. | | | |
| HC3 | **Person to contact**  1 Respondents  2 Family members surveyed  3 Village/neighborhood committee members or village doctors/health service station doctors  4 Neighbor  8 Others, please specify | 1  2  3  4  8 | 1  2  3  4  8 | 1  2  3  4  8 |
| HC4 | Contact results  1 The respondent was contacted  2 No respondents found/contacted  3 The respondent died  9 Others, please specify： | 1  2  3  9 | 1  2  3  9 | 1  2  3  9 |
|  | For the respondents who choose "2" and "9", end the survey.  For the respondents who choose "3", fill in the personal questionnaire: Death DC1-DC6 | | | |

| HC5 | Family questionnaire results  1 Complete  2 Partially completed  3 Refuse to participate  9 Others, please specify： | 1  2  3  9 | 1  2  3  9 | 1  2  3  9 |
| --- | --- | --- | --- | --- |
| HC6 | Follow up investigation results of cardiovascular and cerebrovascular diseases  1 Complete the survey face to face  2 Complete the survey by phone  3Family members complete the survey on their behalf  4 Village/neighborhood committee members or village doctors/health service station doctors complete the investigation on their behalf  5 Partially completed  6 The respondents are not sure whether to accept the survey  7 Respondent refused to investigate | 1  2  3  4  5  6 | 1  2  3  4  5  6 | 1  2  3  4  5  6 |
| HC7 | Follow up survey results of diabetes  1 Complete the survey  2 Partially completed the investigation (only after accepting part of the investigation, the investigation was suspended due to the respondent's refusal or other reasons, and the investigation could not be continued)  3 The respondents are not sure whether to accept the survey  4 Respondent refused to investigate | 1  2  3  4 | 1  2  3  4 | 1  2  3  4 |

The following content is answered by the family members who know the most about the situation.

| Family Questionnaire - Dietary Status |
| --- |

| Family diet | | |
| --- | --- | --- |
| HH1 | a. How many people in your family usually have breakfast at home? | □□people |

|  | b. How many of them are over 2 years old? | □□people |
| --- | --- | --- |
| HH2 | a. How many people in your family usually have lunch at home? | □□people |
|  | b. How many of them are over 2 years old? | □□people |
| HH3 | a. How many people in your family usually have dinner at home? | □□people |
|  | b. How many of them are over 2 years old? | □□people |
| In HH1~HH3, "usually" refers to the most general situation at present. If the working day is different from the weekend situation in the current week, fill in the working day situation. | | |
| HH4 | How many kg of vegetable oil do you usually eat per month?  **Note to investigators**: If you do not know or remember clearly, fill in "- 9" on the right before the decimal point, the same below. | □□. □500g |
| HH5 | How many kg of animal oil do you usually eat in your family? | □□. □500g |
| HH6 | How many kg of salt do you usually eat in a month? | □□. □500g |
| HH7 | How many kg of soy sauce do you usually eat per month? | □□. □500g |
| Rules for filling HH4~HH7: if "do not know", fill in "- 9". | | |

| Family Questionnaire - Economic Status | | |
| --- | --- | --- |
| HH8 | What is your family's total income in 2015?  Note to the investigator: only one item of annual income and monthly income is recorded. | 1 □□□, □□□yuan/month |

|  |  | 2 □□□, □□□yuan/year  99 Don't know the specific income  97 Refuse to answer |
| --- | --- | --- |

二、Individual questionnaire for follow-up survey of cardiovascular and cerebrovascular diseases

Survey start time (24-hour system)：

□□hour □□ minutes

| Part I Basic information | | | | | | | | | | | | | | | | | | | | |
| --- | --- | --- | --- | --- | --- | --- | --- | --- | --- | --- | --- | --- | --- | --- | --- | --- | --- | --- | --- | --- |
| DC | The respondent died? | 1 Yes………………………………………………➔  2 No | | | | | | | | | | | | | | | | | DC1 | |
| A1 | Birthday | Call the data of 2010, without supplementary entry  □□□□year □□month □□ day | | | | | | | | | | | | | | | | | | |
|  | For supplementary entry: the date of birth should be filled in with three items: year, month and day. The date of birth on the ID card should prevail. For example, the date of birth is April 9, 1975, which should be filled in as 1975/4/9.  If the respondents do not remember the date of birth on their ID cards and only remember the lunar birthday, they can postpone the lunar birth date by one month. If the other party's lunar birthday is February 1, 1967, it will be recorded as March 1, 1967.  If you cannot remember the specific year, month and/or day, fill in "- 9" in the corresponding column. | | | | | | | | | | | | | | | | | | | |
| A2 | Gender | Call the data of 2010, without supplementary entry  1 Man  2 Woman | | | | | | | | | | | | | | | | | | |
|  | Select the answer according to the observation, and do not ask the respondents. | | | | | | | | | | | | | | | | | | | |
| A3 | ID number | Some can be imported from the 2011 database, and can be changed if there is an error. If there is no database in 2011, it must be entered | | | | | | | | | | | | | | | | | | |
|  |  |  |  |  |  |  |  |  |  |  |  |  |  |  |  |  |  |  |  |  |
| A4 | Ethic group | 1 Han  7 Gelao  2 Miao  8 Shui  3 Buyi  9 Hui  4 Dong  10 Bai  5 Tujia  11 Other  6 Yi | | | | | | | | | | | | | | | | | | |

| A5 | Your education level | 1 Not receiving formal school education  2 Not graduated from primary school  3 Primary school graduation  4 Junior high school graduation | | 5 High school/technical school  6 Junior college graduation  7 Bachelor degree  8 Postgraduate or above | |
| --- | --- | --- | --- | --- | --- |
|  | Education level: refers to the highest education level obtained by the respondents from domestic and foreign education or the education level equivalent to the existing education level. For the students who have not graduated or have dropped out of school, it refers to the education level that has been obtained. If the respondents are a senior one student, select the option of "junior high school graduation".  1 Not receiving formal school education: refers to the person who has never been to school, or cannot read popular books and newspapers, or write notes.  2 Not graduated from primary school: refers to a person who has received primary education but has not graduated. It also includes people who can read popular books and newspapers, write notes and reach the literacy standard.  3 Primary school graduation: refers to those who have graduated from primary school and have not received junior high school education or above, or have received junior high school education.  4 Junior high school graduation: refers to those who have graduated from junior high school and have not received high school education or above, or have received high school education.  5 Graduates from high school/technical secondary school/technical school: refer to the graduates receiving high school education (including ordinary high school, vocational high school and secondary professional school), as well as the dropouts or current students receiving undergraduate or junior college education.  6 Junior college graduates: refer to those who have received higher education such as national junior college.  7 University graduates: refer to those who have received national undergraduate higher education, and also to those who have been admitted by the state to take the self-taught examination, night university, radio and television university, correspondence university and other universities that have granted undergraduate degrees. As well as those who have received postgraduate education.  8 Postgraduate or above: refers to graduates who have received master's or doctoral education. | | | | |
| A6 | Your current marital status | 1  2  3 | Unmarried  Married  Cohabitation | 4  5  6 | Widow  Divorce  Separation |
|  | Unmarried: refers to those who have never been married.  Married: A person who is in a married state without marital problems, including separation due to work or other reasons.  Cohabitation: cohabitation without marriage.  Widow: Widowed and not remarried.  Divorce: Divorce without remarriage.  Separation: There is a spouse at present, but they live separately from their spouse due to marriage problems. | | | | |
|  |  | | | | |

| A7 | Your occupation | 1 Production personnel of agriculture, forestry, animal husbandry, fishery and water conservancy  2 Production and transportation equipment operators and relevant personnel  3 Business and service personnel  4 Person in charge of state organs, Party mass organizations, enterprises and public institutions  5 Office staff and relevant personnel  6 Professional technicians  7 Solider  8 Other workers  9 Students  10 Unemployed  11 Housework  12 Retired personnel………………………➔ | CA |
| --- | --- | --- | --- |
|  | This includes both employed and non-employed persons. For those who are engaged in several occupations (part-time) at the same time, the occupation with the most fixed working hours and income as the main source of income is the survey object. For those who work again after retirement, if they have worked for more than one year, they will be counted as employees in the current occupation.  Employees:  Production personnel of agriculture, forestry, fishery, animal husbandry and water conservancy: personnel engaged in the production, management and initial processing of products in agriculture, forestry, animal husbandry, fishery and water conservancy.  2.Production and transportation equipment operators and relevant personnel: personnel and relevant personnel engaged in mineral exploration and mining, product production and manufacturing, engineering construction and transportation equipment operation.  3.Business and service personnel: personnel engaged in business, catering, tourism and entertainment, transportation, medical assistance, social and residential life and other services.  4.Heads of state organs, party mass organizations, enterprises and institutions: in the Central Committee of the CPC and local party organizations at all levels, standing committees of people's congresses at all levels, CPPCC, people's courts, people's procuratorates, state administrative organs, democratic parties, trade unions, Communist Youth League, women's federations and other people's organizations, mass autonomous organizations and other mass organizations and their working institutions, enterprises Personnel who hold leading positions and have decision-making and management rights in public institutions.  5 Administrative staff and relevant personnel: personnel engaged in administrative business and administrative affairs in state organs, Party mass organizations, enterprises and institutions, and personnel engaged in security, fire protection, post and telecommunications and other businesses. | | |

|  | 6 Professional and technical personnel: personnel engaged in scientific research and professional and technical work. It includes scientific researchers, scientific and technological management and auxiliary personnel, aircraft and ship technicians, medical and health personnel, legal personnel, economic management professionals, teachers, teaching auxiliary personnel, and literary and sports personnel.  7 Soldiers: refer to the servicemen in the army and the armed police force.  8 Other workers: other employees who cannot be classified.  Non employed personnel:  9 Students at school: refers to the students who are studying in universities and middle schools.  10 Unemployed: refers to students who are unemployed at home and have not found a job after graduation. Retirees are not included.  11 Housework: mainly engaged in household activities, such as laundry, cooking, etc., such as housewives, laid-off workers engaged in household chores at home are unemployed.  12 Retirees: refer to those who have left their posts according to national regulations and have no fixed occupation. | |
| --- | --- | --- |
| **Death information** | | |
| DC1 | Source of death information | 1 Death registration report information system  2 Family members  3 Others, please specify: |
| DA1 | Date of birth | □□□□year □□month □□day  Call the data of 2010, without supplementary entry |
|  | For supplementary entry: the date of birth should be filled in with three items: year, month and day. The date of birth on the ID card should prevail. For example, the date of birth is April 9, 1975, which should be filled in as 1975/4/9.  If the respondents do not remember the date of birth on their ID cards and only remember the lunar birthday, they can postpone the lunar birth date by one month. If the other party's lunar birthday is February 1, 1967, it will be recorded as March 1, 1967.  If you cannot remember the specific year, month and/or day, fill in "- 9" in the corresponding column. | |
| DA2 | Gender | 1 Male  2 Female  Call the data of 2010, without supplementary entry |
|  | Select the answer according to the observation, and do not ask the respondents. | |
| DC2 | Date of death | 1 □□□□year □□month |

|  |  | | 2 Can't remember clearly | | | |
| --- | --- | --- | --- | --- | --- | --- |
|  | Only remember the "year", but not the "month". Fill in "99" for "month" | | | | | |
| DC3 | Root cause of death | | 1. Cardiovascular and cerebrovascular diseases: (fill in the specific disease name)  2.Others: (fill in the specific disease name)  3.Unclear | | | |
| DC4 | IDC10 code | | 1. Cardiovascular and cerebrovascular diseases: (fill in the specific disease name)  2.Others: (fill in the specific disease name)  3.Unclear | | | |
|  | Check the root cause of death and IDC10 code of all dead objects with the cause of death registry. | | | | | |
| DC5 | Diagnostic basis | a. Yes | b．No | | c．Unclear | |
| a | Angiography (DSA) | □ | □ | | □ | |
| b | CT | □ | □ | | □ | |
| c | MR | □ | □ | | □ | |
| d | Physical examination | □ | □ | | □ | |
| e | Operation | □ | □ | | □ | |
| f | Others | □ | □ | | □ | |
| DC6 | Diagnostic unit | | 1 Provincial hospital  2 Municipal hospital  3County level hospital  4 Township level hospitals  5 Other  9 Unclear | | | |
| DC7 | Data sources | | 1 Cause of death registration system  2 Ask family members  3 Others, please specify：............➔ | | | Ending |
| Cardio cerebrovascular disease information | | | | | | |
|  |  | | |  | |  |

| CA | Since 2010, have you been diagnosed with cerebral hemorrhage by doctors? | | 1 Yes  2 No.................……....……........ .....➔ | | CB |
| --- | --- | --- | --- | --- | --- |
| CA0 | Main clinical manifestations | a． Yes | b．No | c．Unclear | |
| a | Sudden headache | □ | □ | □ | |
| b | Nausea and vomiting | □ | □ | □ | |
| c | One limb is weak and feels abnormal | □ | □ | □ | |
| e | Ambiguous or unable to speak | □ | □ | □ | |
| f | Incontinence | □ | □ | □ | |
| g | Unconscious | □ | □ | □ | |
| h | Meningeal irritation sign (such as neck stiffness and lower limb flexion) | □ | □ | □ | |
| i | Dizzy | □ | □ | □ | |
| CA1 | Supplementary examination | a. Yes | b．No | c．Unclear | |
| a | Brain plain CT | □ | □ | □ | |
| b | Brain magnetic resonance imaging (MRI) | □ | □ | □ | |
| c | Transcranial Doppler (TCD) | □ | □ | □ | |
| d | Magnetic resonance angiography (MRA) | □ | □ | □ | |
| e | CT angiography (CTA) | □ | □ | □ | |
| f | Digital subtraction angiography (DSA) | □ | □ | □ | |
| g | Cervical vascular ultrasound | □ | □ | □ | |
| h | Electrocardiogram | □ | □ | □ | |
| i | Echocardiography | □ | □ | □ | |

| CA2 | Time of diagnosis | 1. □□□□year □□month□□day  2. Unclear | |
| --- | --- | --- | --- |
|  | Only remember "year", but not "month" or "day". Fill in "99" for "month" or "day". Is the actual time of onset. | | |
| CA3 | Diagnostic unit | 1 Provincial hospital  2 Municipal hospital  3County level hospital  4 Township level hospitals  5 Other  9 Unclear | |
| CA4 | Is it the first disease since 2010 | 1 Yes ………………………………➔  2 No | CA6 |
| CA5 | Time of second onset (more than 28 days from the first onset since 2010) | 1. Time: □□□□year □□month□□day  2. Unclear | |
|  | If the time of onset is unclear, fill in "- 999" in "year"; Only remember "year", but not "month" or "day". Fill in "99" for "month" or "day". The interval from the first onset is more than 28 days. | | |
| CA5a | Time of the third onset (more than 28 days from the second onset since 2010) | 1 Yes  Time: □□□□year □□month□□day  2 No  3 Unclear | |
|  | If the time of onset is unclear, fill in "- 999" in "year"; Only remember "year", but not "month" or "day". Fill in "99" for "month" or "day". The interval between the second onset and the second onset was more than 28 days. | | |
| CA5b | More than 3 attacks | 1 Yes  2 No  3 Unclear | |
| CA6 | Outcome | 1 Curing  2 Improved  3 Healed | |

|  |  | | 4 Others, please specify： | | |
| --- | --- | --- | --- | --- | --- |
| CB | Since 2010, have you been diagnosed with subarachnoid hemorrhage by your doctor? | | 1 Yes  2 No........……........ ........ .……➔ | | CC |
| CB0 | Clinical symptoms | a. Yes | b. No | c．Unclear | |
| a | Severe headache or vomiting |  | □ | □ | |
| b | Decline in consciousness | □ | □ | □ | |
| c | Meningeal irritation sign | □ | □ | □ | |
| d | Retinal hemorrhage | □ | □ | □ | |
| CB1 | Supplementary examination | a. Yes | b. No | c．Unclear | |
| a | Brain plain CT | □ | □ | □ | |
| b | Brain magnetic resonance imaging (MRI) | □ | □ | □ | |
| c | Transcranial Doppler (TCD) | □ | □ | □ | |
| d | Magnetic resonance angiography (MRA) | □ | □ | □ | |
| e | CT angiography (CTA) | □ | □ | □ | |
| f | Digital subtraction angiography (DSA) | □ | □ | □ | |
| g | Cervical vascular ultrasound | □ | □ | □ | |
| h | Electrocardiogram | □ | □ | □ | |
| i | Echocardiography | □ | □ | □ | |
| j | 24-hour ECG | □ | □ | □ | |
| CB2 | Time of diagnosis | | 1. □□□□year □□month□□day  2. Unclear | | |

|  | Only remember "year", but not "month" or "day". Fill in "99" for "month" or "day". Is the actual time of onset. | | |
| --- | --- | --- | --- |
| CB3 | Diagnostic unit | 1 Provincial hospital  2 Municipal hospital  3County level hospital  4 Township level hospitals  5 Other  9 Unclear | |
| CB4 | Is it the first disease since 2010 | 1 Yes....……...... ..............…➔  2 No | CB6 |
| CB5 | Time of second onset (more than 28 days from the first onset since 2010) | 1. □□□□year □□month□□day  2. Unclear | |
|  | If the time of onset is unclear, fill in "- 999" in "year"; Only remember "year", but not "month" or "day". Fill in "99" for "month" or "day". The interval from the first onset is more than 28 days. | | |
| CB5a | Time of the third onset (more than 28 days from the second onset since 2010) | 1 Yes  Time □□□□year □□month□□day  2 No  3 Unclear | |
|  | If the time of onset is unclear, fill in "- 999" in "year"; Only remember "year", but not "month" or "day". Fill in "99" for "month" or "day". The interval between the second onset and the second onset was more than 28 days. | | |
| CB5b | More than 3 attacks | 1 Yes  2 No  3 Unclear | |
| CB6 | Outcome | 1 Curing  2 Improved  3 Healed | |

|  |  | | 4 Others, please specify： | | |
| --- | --- | --- | --- | --- | --- |
| CC | Since 2010, have you been diagnosed with cerebral infarction by doctors? | | 1 Yes  2 No ..............………..........…➔ | | CD |
| CC0 | Diagnostic basis of main clinical symptoms | a. Yes | b. No | c．Unclear | |
| a | Movement: paralysis involving one or more limbs | □ | □ | □ | |
| b | Feeling: numbness involving one or more limbs | □ | □ | □ | |
| c | Vision: homonymous hemianopia (right eye cannot see things on the right, or left eye cannot see things on the left) | □ | □ | □ | |
| d | Language: Aphasia | □ | □ | □ | |
| CC01 | Secondary clinical symptoms | a. Yes | b. No | c．Unclear | |
| a | Diplopia | □ | □ | □ | |
| b | Vertigo or abnormal balance | □ | □ | □ | |
| c | Dysphagia or dysarthria | □ | □ | □ | |
| d | Severe headache at onset | □ | □ | □ | |
| e | Decline in consciousness | □ | □ | □ | |
| f | Meningeal irritation sign (such as neck stiffness and lower limb flexion) | □ | □ | □ | |
| g | Retinal hemorrhage | □ | □ | □ | |
| h | Paralysis of oculomotor nerve or other nerves | □ | □ | □ | |
| CC1 | Supplementary examination | a. Yes | b. No | c．Unclear | |

| a | Brain plain CT | □ | □ | □ | |
| --- | --- | --- | --- | --- | --- |
| b | Brain magnetic resonance imaging (MRI) | □ | □ | □ | |
| c | Transcranial Doppler (TCD) | □ | □ | □ | |
| d | Magnetic resonance angiography (MRA) | □ | □ | □ | |
| e | CT angiography (CTA) | □ | □ | □ | |
| f | Digital subtraction angiography (DSA) | □ | □ | □ | |
| g | Cervical vascular ultrasound | □ | □ | □ | |
| H | Electrocardiogram | □ | □ | □ | |
| I | Echocardiography | □ | □ | □ | |
| j | 24-hour ECG | □ | □ | □ | |
| CC2 | Time of diagnosis | | 1. □□□□year □□month□□day  2. Unclear | | |
|  | Only remember "year", but not "month" or "day". Fill in "99" for "month" or "day". Is the actual time of onset. | | | | |
| CC3 | Diagnostic unit | | 1 Provincial hospital  2 Municipal hospital  3County level hospital  4 Township level hospitals  5 Other  9 Unclear | | |
| CC4 | Is it the first disease since 2010 | | 1 Yes ……………………………➔  2 No | | CC6 |
| CC5 | Time of second onset (more than 28 days from the first onset since 2010) | | 1. □□□□year □□month□□day  2. Unclear | | |
|  | If the time of onset is unclear, fill in "- 999" in "year"; Only remember "year", but not "month" or "day". Fill in "99" for "month" or "day". The interval from the first onset is more than 28 days. | | | | |

| CC5a | Time of the third onset (more than 28 days from the second onset since 2010) | | 1 Yes  Time □□□□year □□month□□day  2 No  3 Unclear | | |
| --- | --- | --- | --- | --- | --- |
|  | If the time of onset is unclear, fill in "- 999" in "year"; Only remember "year", but not "month" or "day". Fill in "99" for "month" or "day". The interval from the second onset is more than 28 days. | | | | |
| CC5b | More than 3 attacks | | 1 Yes  2 No  3 Unclear | | |
| CC6 | Outcome | | 1 Curing  2 Improved  3 Healed  4 Others, please specify： | | |
| CD | Since 2010, have you been diagnosed with acute myocardial infarction by your doctor? | | 1 Yes  2 No …………………………➔ | | CE |
| CD1 | Diagnostic basis | a. Yes | b. No | c．Unclear | |
| a | Chest pain, sudden onset, long duration, with a sense of dying | □ | □ | □ | |
| b | Electrocardiogram | □ | □ | □ | |
| c | Changes of serum myocardial marker concentration | □ | □ | □ | |
| CD2 | Time of diagnosis | | 1. □□□□year □□month□□day  2. Unclear | | |
|  | Only remember "year", but not "month" or "day". Fill in "99" for "month" or "day". Is the actual time of onset. | | | | |
| CD3 | Diagnostic unit | | 1 Provincial hospital  2 Municipal hospital  3County level hospital  4 Township level hospitals  5 Other | | |

|  |  | 9 Unclear | |
| --- | --- | --- | --- |
| CD4 | Is it the first disease since 2010 | 1 Yes ………………………………➔  2 No | CD6 |
| CD5 | Time of second onset (more than 28 days from the first onset since 2010) | 1. □□□□year □□month□□day  2. Unclear | |
|  | If the time of onset is unclear, fill in "- 999" in "year"; Only remember "year", but not "month" or "day". Fill in "99" for "month" or "day". The interval from the first onset is more than 28 days. | | |
| CD5a | Time of the third onset (more than 28 days from the second onset since 2010) | 1 Yes  Time □□□□year □□month□□day  2 No  3 Unclear | |
|  | If the time of onset is unclear, fill in "- 999" in "year"; Only remember "year", but not "month" or "day". Fill in "99" for "month" or "day". The interval from the second onset is more than 28 days. | | |
| CD5b | More than 3 attacks | 1 Yes  2 No  3 Unclear | |
| CD6 | Outcome | 1 Curing  2 Improved  3 Healed  4 Others, please specify： | |

**Thank you for your cooperation! Survey end time (24-hour system)**： □□Hour□□minutes

3.Personal questionnaire for follow-up survey of blood pressure

According to Article 15 of Chapter III of the Statistics Law of the People's Republic of China, "Individual survey data belonging to private individuals and families shall not be disclosed without their consent".

Chronic disease and nutrition surveillance in Guizhou province

Personal questionnaire for follow-up survey of blood pressure

| Name of respondents: **import** respondents Phone 1: **import** (can be changed or supplemented) | |
| --- | --- |
| Respondent telephone 2 (landline): **import** (can be changed or supplemented) Respondent telephone (mobile phone) 3: **impor**t (can be changed or supplemented) | |
| Contact person of survey object: **import** (can be changed or supplemented) | |
| Contact Phone 1: **Import** (can be changed or added) Contact Phone 2: **Import** (can be changed or added) | |
| Survey point name (county/district): **import** | |
| Township/street name: **import** | |
| Village/neighborhood committee name: **import** | |
| Home address: **import** (can be changed or supplemented) | |
| Investigator Name: | Date: automatically generated |

Note: if the phone number is unknown, fill in-8

Survey start time (24-hour system)：

□□hour □□minutes

| Part I Basic information | | | | | | |
| --- | --- | --- | --- | --- | --- | --- |
| A1 | Birthday | Call the data of 2010, without supplementary entry | | | | |
|  | For supplementary entry: the date of birth should be filled in with three items: year, month and day. The date of birth on the ID card should prevail. For example, the date of birth is April 9, 1975, which should be filled in as 1975/4/9.  If the respondents do not remember the date of birth on their ID cards and only remember the lunar birthday, they can postpone the lunar birth date by one month. If the other party's lunar birthday is February 1, 1967, it will be recorded as March 1, 1967.  If you cannot remember the specific year, month and/or day, fill in "- 9" in the corresponding column. | | | | | |
| A2 | Gender | Call the data of 2010 | | | | |
|  | Select the answer according to the observation, and do not ask the respondents. 1 for males and 2 for females. | | | | | |
| A3 | ID number | Some can be imported from the 2011 database, and can be changed if there is an error. If there is no database in 2011, it must be entered | | |  | |
|  |  |  |  |  |  |  |
| A4 | Ethic group | 1 Han  2 Miao  3 Buyi  4 Dong  5 Tujia  6 Yi | 7 Gelao  8 Shui  9 Hui  10 Bai  11 Other | | | |
| A5 | Your education level | 1 Not receiving formal school education  2 Not graduated from primary school  3 Primary school graduation  4 Junior high school graduation | | 5 High school/technical school  6 Junior college graduation  7 Bachelor degree  8 Postgraduate or above | | |

|  | Education level: refers to the highest education level obtained by the respondents from domestic and foreign education or the education level equivalent to the existing education level. For the students who have not graduated or have dropped out of school, it refers to the education level that has been obtained. If the respondents are a senior one student, select the option of "junior high school graduation".  1 Not receiving formal school education: refers to the person who has never been to school, or cannot read popular books and newspapers, or write notes.  2 Not graduated from primary school: refers to a person who has received primary education but has not graduated. It also includes people who can read popular books and newspapers, write notes and reach the literacy standard.  3 Primary school graduation: refers to those who have graduated from primary school and have not received junior high school education or above, or have received junior high school education.  4 Junior high school graduation: refers to those who have graduated from junior high school and have not received high school education or above, or have received high school education.  5 Graduates from high school/technical secondary school/technical school: refer to the graduates receiving high school education (including ordinary high school, vocational high school and secondary professional school), as well as the dropouts or current students receiving undergraduate or junior college education.  6 Junior college graduates: refer to those who have received higher education such as national junior college.  7 University graduates: refer to those who have received national undergraduate higher education, and also to those who have been admitted by the state to take the self-taught examination, night university, radio and television university, correspondence university and other universities that have granted undergraduate degrees. As well as those who have received postgraduate education.  8 Postgraduate or above: refers to graduates who have received master's or doctoral education. | | | | |
| --- | --- | --- | --- | --- | --- |
| A6 | Your current marital status | 1  2  3 | Unmarried  Married  Cohabitation | 4  5  6 | Widow  Divorce  Separation |
|  | Unmarried: refers to those who have never been married.  Married: A person who is in a married state without marital problems, including separation due to work or other reasons.  Cohabitation: cohabitation without marriage.  Widow: Widowed and not remarried.  Divorce: Divorce without remarriage.  Separation: There is a spouse at present, but they live separately from their spouse due to marriage problems. | | | | |

| A7 | Your occupation | 1 Production personnel of agriculture, forestry, animal husbandry, fishery and water conservancy  2 Production and transportation equipment operators and relevant personnel  3 Business and service personnel  4 Person in charge of state organs, Party mass organizations, enterprises and public institutions  5 Office staff and relevant personnel  6 Professional technicians  7 Solider  8 Other workers  9 Students  10 Unemployed  11 Housework  12 Retired personnel |
| --- | --- | --- |
|  | This includes both employed and non-employed persons. For those who are engaged in several occupations (part-time) at the same time, the occupation with the most fixed working hours and income as the main source of income is the survey object. For those who work again after retirement, if they have worked for more than one year, they will be counted as employees in the current occupation.  Employees:   1. Production personnel of agriculture, forestry, fishery, animal husbandry and water conservancy: personnel engaged in the production, management and initial processing of products in agriculture, forestry, animal husbandry, fishery and water conservancy. 2. Production and transportation equipment operators and relevant personnel: personnel and relevant personnel engaged in mineral exploration and mining, product production and manufacturing, engineering construction and transportation equipment operation.   3. Business and service personnel: personnel engaged in business, catering, tourism and entertainment, transportation, medical assistance, social and residential life and other services.  4. Heads of state organs, party mass organizations, enterprises and institutions: in the Central Committee of the CPC and local party organizations at all levels, standing committees of people's congresses at all levels, CPPCC, people's courts, people's procuratorates, state administrative organs, democratic parties, trade unions, Communist Youth League, women's federations and other people's organizations, mass autonomous organizations and other mass organizations and their working institutions, enterprises Personnel who hold leading positions and have decision-making and management rights in public institutions.  5. Administrative staff and relevant personnel: personnel engaged in administrative business and administrative affairs in state organs, Party mass organizations, enterprises and institutions, and personnel engaged in security, fire protection, post and telecommunications and other businesses.  6. Professional and technical personnel: personnel engaged in scientific research and professional and technical work. It includes scientific researchers, scientific and technological management and auxiliary personnel, aircraft and ship technicians, medical and health personnel, legal personnel, economic management professionals, teachers, teaching auxiliary personnel, and literary and sports personnel.  7. Soldiers: refer to the servicemen in the army and the armed police force.  8. Other workers: other employees who cannot be classified. | |

|  | Non employed personnel:  9 Students at school: refers to the students who are studying in universities and middle schools.  10 Unemployed: refers to students who are unemployed at home and have not found a job after graduation. Retirees are not included.  11 Housework: mainly engaged in household activities, such as laundry, cooking, etc., such as housewives, laid-off workers engaged in household chores at home are unemployed.  12 Retirees: refer to those who have left their posts according to national regulations and have no fixed occupation. | | |  |
| --- | --- | --- | --- | --- |
| Part II Weight, blood pressure, blood sugar, blood lipid and other information | | | | |
| Weight and its control | | | | |
| C1 | | What do you think of your current weight? | 1 Thin  2 Normal  3 Overweight  4 Obesity | |

| C2 | Have you taken the following measures to control or reduce weight? | | 1 Don’t take measures to control weight………➔  2 Taking measures to control weight | | | C3 |
| --- | --- | --- | --- | --- | --- | --- |
|  | Taking measures refers to a series of behaviors that are planned, active and aimed at weight control. The duration should not be less than 1 week. | | | | | |
| a | Diet control | | 1 Yes  2 No | | | |
| b | Physical exercise | | 1 Yes  2 No | | | |
| c | Medicine | | 1 Yes  2 No | | | |
| d | Other | | 1 Yes, please specify：  2 No | | | |
| Diet control: reduce the intake of carbohydrates and lipids, increase the intake of vegetables and fruits, and adjust the dietary structure;  Exercise: It mainly refers to regular physical exercise, which can’t be counted as once in a while;  Medication: take the medicine according to the doctor's advice after going to the doctor, or buy the medicine to control the weight;  All measures should take weight control as the ultimate goal. If the subsidiary behavior of other purposes cannot be considered as the method of weight control. | | | | | | |
| Blood pressure and its control | | | | | | |
| C3 | Do your following relatives have high blood pressure? | a. Yes | | b. No | c. Unclear | |
| a | Grandparents | □ | | □ | □ | |
| b | Parents | □ | | □ | □ | |
| c | Brother/Sister | □ | | □ | □ | |
| It must be diagnosed by the doctors in the township health center or community health service center or above. Only immediate relatives are considered, not cousins. | | | | | | |
| C4 | Have you been diagnosed with hypertension by a doctor **in a township health center or community health service center or above**? | | 1 Yes，  **First diagnosis** at □□□□year □□month or □□year  2 No…………………………………➔ | | | C7 |

|  | It must be diagnosed by the doctors in the township health center or community health service center or above. If answer 2 "No", go to D7. | | |
| --- | --- | --- | --- |
| C5 | Have you taken the following measures to control your blood pressure? | 1No……………………………………➔  2 Yes | C7 |
| a | Insist on taking antihypertensive drugs for a long time according to the doctor's advice | 1 Yes  2 No | |
| b | Take antihypertensive drugs in stages | 1 Yes  2 No | |
| c | Take medicine when blood pressure is high | 1 Yes  2 No | |
| d | Diet control | 1 Yes  2 No | |
| e | Physical exercise | 1 Yes  2 No | |
| f | Blood pressure monitoring | 1 Yes  2 No | |
| g | Other | 1 Yes, please specify：  2 No | |
| C6 | Have you taken any antihypertensive drugs in the last 2 weeks? | 1 Yes  2 No | |
|  | To understand the situation of hypertension patients taking antihypertensive drugs in the past 2 weeks. | | |
| Blood lipid and its control | | | |
| C7 | Have you ever been diagnosed as dyslipidemia or hyperlipidemia by doctors **in township health centers or community health service centers or medical institutions at or above** the level? | 1 Yes，  **First diagnosis** at □□□□year □□month or □□ year  2 No…………………………………➔ | C10 |
|  | It must be diagnosed by the doctors in the township health center or community health service center or above. | | |

| C8 | What are the types of dyslipidemia? | 1. Yes | | 1. No | | 1. Unclear | | |
| --- | --- | --- | --- | --- | --- | --- | --- | --- |
| a | Hypercholesterolemia | □ | | □ | | □ | | |
| b | Hypertriglyceridemia | □ | | □ | | □ | | |
| c | Low density lipoproteinemia | □ | | □ | | □ | | |
| d | Low high-density lipoproteinemia | □ | | □ | | □ | | |
| C9 | What measures have you taken to control blood lipids? | | | 1 Don’t take measures to control blood lipid ……………………➔  2 Take measures to control blood lipid | | | | C10 |
| a | Take medicine for a long time according to the doctor's advice | | | 1 Yes  2 No | | | | |
| b | Take blood lipid lowering drugs in stages | | | 1 Yes  2 No | | | | |
| c | Diet control | | | 1 Yes  2 No | | | | |
| d | Physical exercise | | | 1 Yes  2 No | | | | |
| e | Blood lipid monitoring | | | 1 Yes  2 No | | | | |
| f | Other | | | 1 Yes, please specify：  2 No | | | | |
| Blood glucose and its control | | | | | | | | |
| C10 | Do your following relatives have diabetes? | | d. Yes | | e. No | | f. Unclear | |
| a | Grandparents | | □ | | □ | | □ | |

| b | Parents | □ | | □ | □ | |
| --- | --- | --- | --- | --- | --- | --- |
| c | Brother/Sister | □ | | □ | □ | |
| It must be diagnosed by the doctors in the township health center or community health service center or above. Only immediate relatives are considered, not cousins. | | | | | | |
| C11 | Have you ever been diagnosed as diabetes by a doctor **in a township health center or community health service center or a medical institution at or above** the level? | | 1 Yes，  **First diagnosis** at □□□□year □□month or □□ year  2 No…………………………………➔ | | | Ending |
|  | It must be diagnosed by the doctors in the township health center or community health service center or above. | | | | | |
| C12 | What measures have you taken to control blood sugar? | | 1 Don’t take measures ……………………➔  2 Take measures to control blood sugar | | | Ending |
| a | Oral medicine | | 1 Yes  2 No | | | |
| b | Insulin injection | | 1 Yes  2 No | | | |
| c | Diet control | | 1 Yes  2 No | | | |
| d | Physical exercise | | 1 Yes  2 No | | | |
| e | Blood glucose monitoring | | 1 Yes, please specify：  2 No | | | |
| f | Other | |  | | | |

Thank you for your cooperation! Survey end time (24-hour system)： □□hour □□minutes

Part III Body Measurement Record

| Personal code | Direct import |
| --- | --- |

| Hello, now we will measure your weight, waist circumference and blood pressure. Please cooperate. | | | |
| --- | --- | --- | --- |
| M1a | Name of surveyor 1 |  | |
| M1b | Name of surveyor 2 |  | |
| M2 | Weight  Note to the investigator: if the weight exceeds the range, record - 9. | □□□. □kilogram (kg) | |
| M3 | Height  Note to the investigator: if the height exceeds the range, record - 9 | □□□. □centimeter (cm) | |
| M4 | Waist | □□□. □centimeter (cm) | |
|  |  | □□□. □centimeter (cm) | |
| Blood pressure | | | |
| M5 Electronic sphygmomanometer | Name of surveyor | M6 indoor temperature □□℃ | |
| M5a | First reading  Note to the investigator: measure and record the blood pressure for the first time after 15 minutes of rest, and measure the blood pressure for the second time after 1 minute of rest. | Systolic pressure | □□□ (mmHg) |
| M5b |  | Diastolic pressure | □□□ (mmHg) |
| M6a | Second reading | Systolic pressure | □□□ (mmHg) |

| M6b | Record the second measurement result. | Diastolic pressure | □□□ (mmHg) |
| --- | --- | --- | --- |
| M7a | Third reading  Record the third measurement result. | Systolic pressure | □□□ (mmHg) |
| M7b |  | Diastolic pressure | □□□ (mmHg) |
| M8 Mercury sphygmomanometer | Name of surveyor |  | |
| M8a | First reading  Note to the investigator: measure and record the blood pressure for the first time after 15 minutes of rest, and measure the blood pressure for the second time after 1 minute of rest. | Systolic pressure | □□□ (mmHg) |
| M8b |  | Diastolic pressure | □□□ (mmHg) |
| M9a | Second reading  Record the second measurement result. | Systolic pressure | □□□ (mmHg) |
| M9b |  | Diastolic pressure | □□□ (mmHg) |
| M10a | Third reading  Record the third measurement result. | Systolic pressure | □□□ (mmHg) |
| M10b |  | Diastolic pressure | □□□ (mmHg) |
